# Identification of miRNA Biomarkers for Diverse Cancer Types Using Statistical Learning Methods at the Whole-Genome Scale

**DOI:** 10.3389/fgene.2020.00982

**Published:** 2020-11-13

**Authors:** Jnanendra Prasad Sarkar, Indrajit Saha, Adrian Lancucki, Nimisha Ghosh, Michal Wlasnowolski, Grzegorz Bokota, Ashmita Dey, Piotr Lipinski, Dariusz Plewczynski

**Affiliations:** ^1^Data, Analytics & AI, Larsen & Toubro Infotech Ltd., Pune, India; ^2^Department of Computer Science & Engineering, Jadavpur University, Kolkata, India; ^3^Department of Computer Science and Engineering, National Institute of Technical Teachers' Training and Research, Kolkata, India; ^4^Computational Intelligence Research Group, Institute of Computer Science, University of Wroclaw, Wroclaw, Poland; ^5^Department of Computer Science and Information Technology, SOA University, Bhubaneshwar, India; ^6^Faculty of Mathematics and Information Science, Warsaw University of Technology, Warsaw, Poland; ^7^Institute of Informatics, University of Warsaw, Warsaw, Poland; ^8^Centre of New Technologies, University of Warsaw, Warsaw, Poland

**Keywords:** cancer, cox regression, feature selection, gene ontology, KEGG pathway, machine learning, next generation sequencing, stochastic neighbor embedding

## Abstract

Genome-wide analysis of miRNA molecules can reveal important information for understanding the biology of cancer. Typically, miRNAs are used as features in statistical learning methods in order to train learning models to predict cancer. This motivates us to propose a method that integrates clustering and classification techniques for diverse cancer types with survival analysis via regression to identify miRNAs that can potentially play a crucial role in the prediction of different types of tumors. Our method has two parts. The first part is a feature selection procedure, called the stochastic covariance evolutionary strategy with forward selection (SCES-FS), which is developed by integrating stochastic neighbor embedding (SNE), the covariance matrix adaptation evolutionary strategy (CMA-ES), and classifiers, with the primary objective of selecting biomarkers. SNE is used to reorder the features by performing an implicit clustering with highly correlated neighboring features. A subset of features is selected heuristically to perform multi-class classification for diverse cancer types. In the second part of our method, the most important features identified in the first part are used to perform survival analysis via Cox regression, primarily to examine the effectiveness of the selected features. For this purpose, we have analyzed next generation sequencing data from The Cancer Genome Atlas in form of miRNA expression of 1,707 samples of 10 different cancer types and 333 normal samples. The SCES-FS method is compared with well-known feature selection methods and it is found to perform better in multi-class classification for the 17 selected miRNAs, achieving an accuracy of 96%. Moreover, the biological significance of the selected miRNAs is demonstrated with the help of network analysis, expression analysis using hierarchical clustering, KEGG pathway analysis, GO enrichment analysis, and protein-protein interaction analysis. Overall, the results indicate that the 17 selected miRNAs are associated with many key cancer regulators, such as MYC, VEGFA, AKT1, CDKN1A, RHOA, and PTEN, through their targets. Therefore the selected miRNAs can be regarded as putative biomarkers for 10 types of cancer.

## 1. Introduction

MicroRNAs (miRNAs) belong to the non-coding RNA family. They consist of 19–25 nucleotides and play an important role in the regulation of gene silencing. These non-coding RNAs are present in every eukaryotic cell and can also be encoded by a viral genome (Ray and Maiti, 2015; Bruscella et al., 2017). The miRNAs are formed by RNA polymerase II in the cell nucleus and are then transferred to the cytoplasm (Bartel, 2009) for biological activities such as cell cycle control, apoptosis, and oncogenesis. They interact with the complementary strand of mRNAs and lead to the degradation of the corresponding mRNAs; they also interfere with protein production by suppressing protein synthesis (Valencia-Sanchez et al., 2006). A miRNA molecule can bind one or more targets, thus forming a complex underlying regulatory network. These networks have a profound impact on cancer signaling pathways (Wang et al., 2017). Previously, low-throughput and high-cost technologies were the main obstacle to answering systems-level biological questions. However, recent advancements in next generation sequencing (NGS) have enabled researchers to address such complex problems (van Dijk et al., 2014). Moreover, new sequencing technologies and genomic datasets have helped us to gain better understanding of the biological complexities related to genomic abnormalities in cancer. The considerable achievements in sequencing techniques have made high-throughput techniques a fundamental platform for miRNA, RNA, and DNA research. Generally, miRNAs are involved in a wide range of diseases, including neurological disease, heart disease, and cancer (Giza et al., 2014; Paul et al., 2018). In many cases of cancer in humans, dysregulation of miRNA expression has been observed, and it is well-known that miRNAs can serve as potential cancer biomarkers (Lu et al., 2005; Jacobsen et al., 2013; Wong et al., 2017). In this regard, scientific communities are also trying to understand the role of miRNAs in paring with mRNAs (Zhang et al., 2014; Shrestha et al., 2017), in different cancer types by ranking miRNAs (Li et al., 2014) to elucidate their effects and drug resistance (Ma et al., 2010; Li and Yang, 2014; Cheerla and Gevaert, 2017).

To reduce the time taken for clinical trials, and to provide better and more accurate treatments while avoiding unnecessary interventions, the proper selection of miRNAs as biomarkers is crucial. For this purpose, miRNAs are often used as features in statistical learning methods viz. clustering, classification, and regression in order to identify potential biomarkers (Song et al., 2016; Yang et al., 2017; Yokoi et al., 2017). Song et al. (2016) performed a clustering analysis on breast cancer data in order to find miRNAs that could be prognostic biomarkers; these miRNAs are up-regulated in this type of cancer and are linked to local relapse, distant metastasis, and poor clinical outcomes. Similarly, Yang et al. (2017) used clustering to find miRNA biomarkers for breast cancer. The identified miRNAs have higher specificity and sensitivity than single-gene biomarkers. On the other hand, Yokoi et al. (2017) used a classification task to inform the development of a predictive model to distinguish patients with ovarian cancer tumors from healthy subjects. In this study, eight miRNAs were found as biomarkers for ovarian cancer. Jacob et al. (2017) conducted a study on colon cancer and identified 16 miRNA signatures, which act as prognostic biomarkers at cancer stages II and III. Apart from the aforementioned works, regression analysis has been used to predict the survival rates of patients with different types of cancer (Liang et al., 2018). Liang et al. (2018) used Cox regression analysis for pancreatic cancer and identified five miRNAs as independent prognostic factors. The progress in this regard can be found in the literature (e.g., Peng and Croce, 2016; Hosseinahli et al., 2018).

Generally, in miRNA-based cancer studies, statistical learning algorithms viz. clustering, classification, and regression are used separately for different cancer types, as described above and in the literature (Akhtar et al., 2015; Ang et al., 2016; Li et al., 2016; Lin and Lane, 2017). However, to leverage the advantages of the different algorithms, it may be useful to integrate them into a single method for identifying potential biomarker miRNAs. Besides, little work exists on multi-class classification of diverse cancer types using NGS data. These two facts motivated us to develop the method described in this paper, which can not only classify 10 types of cancer (bladder, breast, colon, glioblastoma, head and neck squamous cell, kidney renal clear cell, lung adenocarcinoma, lung squamous cell, ovarian, and uterine corpus endometrial carcinoma) but also find putative miRNAs that are highly associated with these cancer types. The proposed two-part wrapper-based feature selection method, referred to as the stochastic covariance evolutionary strategy with forward selection (SCES-FS), uses stochastic neighbor embedding (SNE) (Hinton and Roweis, 2003) in conjunction with the covariance matrix adaptation evolutionary strategy (CMA-ES) (Hansen et al., 2003) and a simple classifier, either random forest (RF) (Breiman, 2001), support vector machine (SVM) (Cortes and Vapnik, 1995), naive Bayes (NB) classifier (George and Langley, 1995), *K*-nearest-neighbors (*K*-NN) classifier (Altman, 1992), or decision tree (DT) (Quinlan, 1986), in the first part. Here SNE is used to reorder the features by performing an implicit clustering, such that neighboring features are highly correlated. Then, from these clusters of features, a subset of features is selected randomly in order to perform the multi-class classification task for the diverse cancer types. However, as the features are randomly selected, this classification task is treated as an underlying optimization problem for CMA-ES to find the features automatically. Hence, the final set of features/miRNAs is obtained by using forward selection (Whitney, 1971).

In the second part of the method, survival analysis is performed using Cox regression (Cox, 1972), where the levels of miRNA expression and corresponding clinical data are used. The experiment is conducted with data collected from The Cancer Genome Atlas (TCGA)^1^ for 10 different types of cancer. The performance of the proposed wrapper-based feature selection method is compared with the following methods in terms of classification accuracy, with the top 17 miRNAs selected as putative biomarkers: ensemble SVM-recursive feature elimination (ESVM-RFE) (Anaissi et al., 2016), the least absolute shrinkage and selection operator (LASSO) (Tibshirani, 1996), the non-dominated sorting genetic algorithm II-based stacked ensemble (NSGA-II-SE) (Saha et al., 2017), the SVM-wrapped multi-objective genetic algorithm (MOGA) (Mukhopadhyay and Maulik, 2013), SVM-based novel recursive feature elimination (SVM-nRFE) (Peng et al., 2009), SVM recursive feature elimination (SVM-RFE) (Guyon et al., 2002), conditional mutual information (CMIM) (Fleuret, 2004), interaction capping (ICAP) (Jakulin, 2005), smoothly clipped absolute deviation (SCAD) (Fan and Li, 2001), joint mutual information (JMI) (Bennasar et al., 2015), conditional infomax feature extraction (CIFE) (Brown et al., 2012), minimum redundancy maximum relevance (mRMR) (Peng et al., 2005), feature selection with Cox regression (FSCOX) (Kim et al., 2014), double-input symmetrical relevance (DISR) (Brown et al., 2012), signal-to-noise ratios (SNRs) (Mishra and Sahu, 2011), and the Wilcoxon rank-sum test (RankSum) (Troyanskaya et al., 2002). Thereafter, the significance of the 17 selected miRNAs to 10 different cancer types is determined using Cox regression analysis. Finally, survival analysis, network analysis, expression analysis using hierarchical clustering in the form of heatmaps, KEGG pathway analysis (Kanehisa and Goto, 2000), gene ontology (GO) enrichment analysis (Kuleshov et al., 2016), and protein-protein interaction (PPI) network analysis (Szklarczyk et al., 2019) are performed to assess the biological significance of the selected miRNAs. Additionally, a web-based cancer predictor application is developed to predict 10 different types of cancer given the expression of 17 miRNAs.

## 2. Materials and Methods

**Figure 1:**
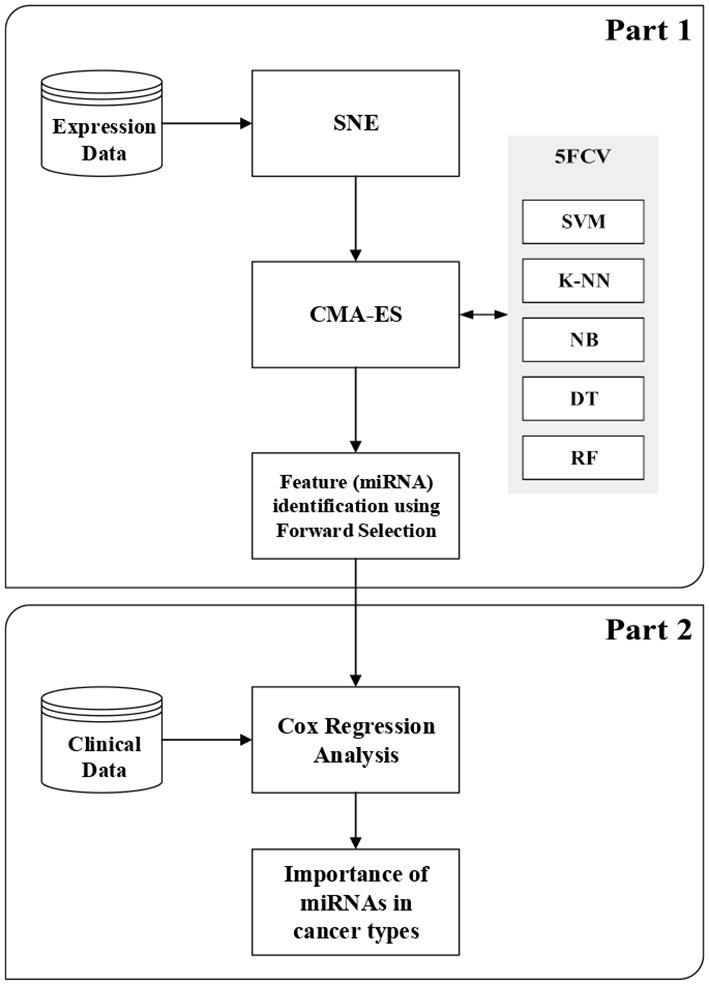
Flowchart for the proposed method.

In this section, we briefly describe SNE (Hinton and Roweis, 2003), CMA-ES (Hansen, 2006), and Cox regression analysis (Cox, 1972). More details about classification techniques and feature selection methods are given in the Supplementary Material^2^ for this article. This section also describes the proposed method, which consists of two parts. The first part is the wrapper-based SCES-FS. In the second part, the selected features, such as expression of miRNAs and clinical data, are used in survival analysis to assess the importance of the selected miRNAs in different types of cancer and to evaluate the effectiveness of the selected miRNAs. Figure 1 shows the flowchart of the proposed method.

### 2.1. Stochastic Neighbor Embedding

Let $X=\left\{{\text{x}}_{1},x_{2},\ldots ,x_{n}\right\}$ denote a set of *n* observations, where $x_{i}\in {\mathbb{R}}^{D}$. SNE (Hinton and Roweis, 2003) constructs a low-dimensional embedding that recreates X in a space of lower dimension as $X^{\prime }=\{{x^{\prime }}_{1},{x^{\prime }}_{2},\ldots ,{x^{\prime }}_{n}\}$, where $x_{i}^{\prime }\in {\mathbb{R}}^{d}$. In SNE, both X and ${X^{\prime }}_{\rangle }$ are represented as discrete probability distributions *P* and *Q*, where

(1) $$\begin{array}{l} p_{ij}=\frac{\exp\left(\frac{-∥x_{i}-x_{j}{∥}^{2}}{2\text{va}{\text{r}}_{i}}\right)}{\sum_{k\neq i}\exp\left(\frac{-∥x_{i}-x_{k}{∥}^{2}}{2\text{va}{\text{r}}_{i}}\right)},\;q_{ij}=\frac{\exp\left(-∥x_{i}^{\prime }-x_{j}^{\prime }{∥}^{2}\right)}{\sum_{k\neq l}\exp\left(-∥x_{l}^{\prime }-x_{k}^{\prime }{∥}^{2}\right)}, \end{array}$$

that model pairwise distances between data points. The values of var_*i*_ ∈ ℝ are adjusted in such a way that the entropies of all distributions *P*_*i*_ are equal.

The mismatch between *P* and *Q* is reduced through minimization of Kullback-Leibler (KL) divergence objective $\text{KL}\left(P||Q\right)=\underset{i}{\sum }\underset{j}{\sum }p_{ij}\log\frac{p_{ij}}{q_{ij}}=\underset{i}{\sum }\text{KL}\left(P_{i}||Q_{i}\right)$, by altering *Q* with gradient-based optimization methods. Optimization is difficult due to the existence of multiple local optima, and entirely different embeddings may be obtained with different initial *Q* distributions.

### 2.2. Covariance Matrix Adaptation Evolution Strategy

Evolutionary strategies are black-box optimization algorithms which belong to a broader group of evolutionary algorithms. In such methods, a set of candidate solutions is maintained. In successive iterations of the procedure, these candidate solutions are perturbed and evaluated, and in each iteration the best solution is left unchanged and carried over to the next set of candidate solutions. CMA-ES (Hansen and Ostermeier, 1996) is an evolutionary strategy where the set of candidate solutions is modeled and sampled from a multivariate Gaussian distribution N(m,C). The covariance matrix **C** represents pairwise relationships between attributes. The objective function of CMA-ES maximizes two likelihoods: (a) the likelihood of having the best individuals in previous iterations, and (b) the likelihood of taking the best search steps in previous iterations. At the end of each iteration, (a) guides updates of the mean **m** as a weighted average of μ best solutions, ${\text{m}}^{\left(g+1\right)}=\overset{\mu }{\underset{i=0}{\sum }}w_{i}x_{i}^{\left(g+1\right)},$ where $x_{i}^{\left(g+1\right)}$ is the *i*th best solution in iteration *g* + 1 and *w*_*i*_ is its weight, and (b) updates the covariance matrix **C** as follows:

(2) $$\begin{array}{l} {\text{C}}^{\left(g+1\right)}=\left(1-c_{1}-c_{\mu }\right){\text{C}}^{\left(g\right)}+c_{1}{\text{p}}_{c}^{\left(g+1\right)}{\text{p}}_{c}^{{\left(g+1\right)}^{T}}\\ \;+c_{\mu }\overset{\mu }{\underset{i=1}{\sum }}w_{i}\left(\frac{x_{i}^{\left(g+1\right)}-{\text{m}}^{\left(g\right)}}{\sigma^{\left(g\right)}}\right){\left(\frac{x_{i}^{\left(g+1\right)}-{\text{m}}^{\left(g\right)}}{\sigma^{\left(g\right)}}\right)}^{T}. \end{array}$$

Finally, ${\text{p}}_{c}^{\left(g+1\right)}\in {\mathbb{R}}^{D}$ is a vector that amplifies the updates in favorable directions:

(3) $$\begin{array}{l} {\text{p}}^{\left(g+1\right)}=\left(1-c_{c}\right){\text{p}}_{c}^{\left(g\right)}+\;z\frac{{\text{m}}^{\left(g+1\right)}-{\text{m}}^{\left(g\right)}}{\sigma^{\left(g\right)}}, \end{array}$$

where *c*_1_, *c*_μ_, *c*_*c*_ ∈ ℝ are weights, σ^(*g*)^ ∈ ℝ is an adaptive step size, which is dependent on the iteration, and *z* ∈ ℝ is a normalizing constant. Details of the parameters of CMA-ES can be found in Hansen and Ostermeier (1996) and Hansen et al. (2003).

### 2.3. Cox Regression Analysis

The Cox regression model (Cox, 1972) is a proportional hazards regression model in which the hazard ratio is constant but other contents have the same baseline hazard function. Based on this assumption, the survival function is calculated as

(4) $$\begin{array}{l} S\left(\tau \right)=\exp\left(-H_{0}\left(\tau \right)\exp\left(X\beta \right)\right)=S_{0}{\left(\tau \right)}^{\exp\left(X\beta \right)}, \end{array}$$

where *H*_0_(τ) represents the cumulative baseline hazard function at time τ and *S*_0_(τ) = exp(−*H*_0_(τ)) is the baseline survival function; *H*_0_(τ) is taken to be Breslow's estimator (Breslow, 1974), which is the most widely used and given by

(5) $$\begin{array}{l} {\hat{H}}_{0}\left(\tau \right)=\underset{\tau_{i}\leq \tau }{\sum }{\hat{h}}_{0}\left(\tau_{i}\right). \end{array}$$

As the Cox model is based on the proportional hazards assumption, it is represented as

(6) $$\begin{array}{l} h\left(\tau ,x_{i}\right)=h_{0}\left(\tau \right)\exp\left(x_{i}\beta \right) \end{array}$$

for an given instance *i* = 1, 2, 3, …, *n*, where the baseline hazard function *h*_0_(τ) can be an arbitrary negative function of time, and *x*_*i*_ = (*x*_*i*1_, *x*_*i*2_, …, *x*_*iD*_) is the corresponding covariate vector for instance *i* and is the coefficient vector. The Cox model is a semi-parametric algorithm where the baseline hazard function *h*_0_(τ) is unspecified. For any two instances *x*_1_ and *x*_2_, the hazard ratio is given by

(7) $$\begin{array}{l} \frac{h\left(\tau ,x_{1}\right)}{h\left(\tau ,x_{2}\right)}=\frac{h_{0}\left(\tau \right)\exp\left(x_{1}\beta \right)}{h_{0}\left(\tau \right)\exp\left(x_{2}\beta \right)}=\exp\left[\left(x_{1}-x_{2}\right)\beta \right]. \end{array}$$

This means that the hazard ratio is independent of the baseline hazard function.

### 2.4. Wrapper-Based Feature Selection Integrating SNE and CMA-ES

The first part of our proposed method performs the task of miRNA selection for diverse cancer types, which is considered a multi-class classification problem here. SNE is used to reorder the features by performing an implicit clustering such that neighboring features are highly correlated. Then, the underlying multi-class classification task is performed using well-known classifiers and treated as an optimization problem for which CMA-ES is used to find the miRNAs automatically. The miRNAs thus found are further refined using forward selection (FS). Therefore, we call this wrapper-based feature selection method as stochastic covariance evolutionary strategy with forward selection (SCES-FS).

Algorithm 1 presents the SCES-FS method in detail. It starts with the dataset $X=\left\{x_{1},x_{2},\ldots ,x_{n}\right\}$, which denotes a set of *n* observations with $x_{i}\in {\mathbb{R}}^{D}$, the class label Y, the population size λ, the maximum number of generations *N*, the classifier A, and the number of runs R as inputs. In the dataset, each feature is characterized by expression levels of the samples. The features of the original dataset X are reordered using SNE in the *ConstructEmbedding* step, producing the embedding dataset $X^{\prime }$ whose size is the same as that of the original dataset. The parameters are initialized in the *SetParamsCMAES* step. The individuals/vectors in CMA-ES are encoded as a simple threshold weight vector, with a single weight for each miRNA. An individual *x* ∈ ℝ^*H*^ encodes a weight vector *w* ∈ ℝ^*H*^ and a threshold *t* ∈ ℝ, where *H* ≤ *D* is the number of weights. Only those features whose weights exceed the threshold are eventually selected into a feature set *S*:

(8) $$\begin{array}{l} S=\left\{i\in 1,\ldots ,D:\text{closest}\left(x_{i}\right)=j\wedge w_{i}\geq t\right\}, \end{array}$$

where *t* ∈ ℝ is a threshold that is chosen carefully. The population of vectors is drawn in the *DrawPopulationCMAES* step from $N\left(\text{m},\sigma^{2}\text{C}\right)$, where **m** is the mean, **C** is the covariance matrix, and σ is the step-size control parameter of CMA-ES. In the *ConstructFeatureSets* step, each individual *x*_*i*_ is translated to a feature set *S*_*i*_ according to Equation (8).

**Algorithm 1:** Pseudo-code of the SCES-FS

**Input:** X,Y,λ,N,A,R (dataset, class label, population size, maximum number of generations, classifier, number of runs)
**Output:** *S*_best_ (feature subset)
1: Initialize a NULL list, *L*
2: **for** *i* ← 1 **to** R **do**
3: $X^{\prime }$ ← *ConstructEmbedding* (X) // With SNE
4: θ ← *SetParamsCMAES()* // μ, σ, **m**, **C**
5: **for** *g* ← 1 **to** *N* **do**
6: P ← *DrawPopulationCMAES* ($X^{\prime },\text{ λ},\theta$)
7: S←ConstructFeatureSets(P)
8: $\text{ScoreFeatureSet}\left(S,X^{\prime },A,Y\right)$
9: $S_{\text{RBest}}\leftarrow \text{BestFeatureSet}\left(S,S_{\text{RBest}}\right)$
10: θ ← *UpdateParamsCMAES*(θ)
11: **end** **for**
12: *L* ← *L* ∪ *S*_RBest_
13: **end** **for**
14: *L'* ← *RankFrequency*(*L*)
15: $S_{\text{best}}\leftarrow \text{ForwardSelection}\left(\text{L'},X^{\prime },A,Y\right)$
16: **return** *S*_best_

In the *ScoreFeatureSet* step, each feature set is evaluated by training a classifier on a dataset restricted to only the selected features. The objective function of CMA-ES combines the accuracy obtained from the classifier for each individual *x* and the size of the feature set *S* as follows:

(9) $$\begin{array}{l} f\left(x\right)=\text{accuracy}\left(S\right)-\alpha \frac{\max\left(0,|S|-\Upsilon \right)}{D}, \end{array}$$

where *D* is the dimension of the dataset, Υ is the target number of features, and α ∈ ℝ_+_ balances the penalty for the excessive number of features. The objective function is designed in this way so that higher classification accuracy can be achieved for a small number of features, and it incorporates *L*_0_ regularization term as penalty, which is bounded by Υ in order to have redundancy in selected subsets of features. The optimization with CMA-ES allows inter-feature relationships with covariance matrices. Finally, the parameters are updated according to CMA-ES update rules in the *UpdateParamsCMAES* step, and the best set of features/miRNAs in a particular run are kept in *S*_RBest_.

### 2.5. Preparation of the Final Set of Features

The SCES-FS is random in nature to reduce the probability of returning sub-optimal solutions. Therefore, a single run of SCES-FS does not guarantee a reliable solution. To overcome this limitation, SCES-FS is executed up to a maximum number of runs, R. In each run, a set of best features/miRNAs are collected into a list, *L*. After completion of the maximum number of runs, *RankFrequency* sorts the cumulative set of features in descending order according to the frequency of occurrence in each run and produces a modified list, *L*′. Thereafter, *ForwardSelection* applies the forward feature selection method, using the classifier to evaluate the feature set iteratively to obtain the best feature set, *S*_best_.

### 2.6. Justification for miRNA Selection

Because of the random nature of the algorithm, there is a chance of false positives or false negatives occurring in the selection of miRNAs. To reduce the probability of having false positives or false negatives in the selected set of miRNAs, the SCES-FS algorithm is run 50 times, and then the miRNAs are ranked based on their frequencies of occurrence in 50 different sets of features. Thus, a stable set of miRNAs is selected on the basis of maximum classification accuracy, which is computed by considering the miRNAs cumulatively from the top of the list. By this process, 17 distinct miRNAs are selected.

This procedure does not, however, ensure the absence of false positives or false negatives, so additional measures are taken. The presence of false positives is made unlikely by the sorting procedure. In fact, since a given false positive is supposed to occur less frequently than all the true positives, it will appear in the tail after sorting. Consequently, it is likely that this false positive will be filtered out when selecting the final list of miRNAs. On the other hand, the presence of false negatives is related to the choice of the number of runs and the corresponding expected number of miRNAs belonging to the sorted list. In this regard, a mathematical argument is given below to justify that the SCES-FS does not exhibit random behavior.

Suppose that at each run the SCES-FS randomly selects 10 miRNAs from the whole collection of miRNAs. We first compute the expected number of distinct miRNAs reported after all the runs, and then we compare this number with the results of our experiments. We have the following parameters: *D* = 199 is the number of miRNAs, *S* = 10 is the number of miRNAs returned after each run, and R=50 is the number of runs. For *i* = 1, …, *D*, let *V*_*i*_ be a Bernoulli-distributed indicator variable, where *V*_*i*_ = 1 if the miRNA *m*_*i*_ never shows up. The probability that *m*_*i*_ is selected in one run is *S*/*D*, so the probability that it is never selected is 1−*S*/*D*. Since each of the R runs is independent, the following equation can be written:

(10) $$\begin{array}{l} E\left[V_{i}\right]=\text{Pr}\left(V_{i}=1\right)={\left(1-\frac{S}{D}\right)}^{R}. \end{array}$$

Let $V=\overset{D}{\underset{i=1}{\sum }}V_{i}$ be the random variable that counts the number of miRNAs that do not belong to the final set of miRNAs reported at least once. By linearity of the expectation, we obtain the equation

(11) $$\begin{array}{l} E\left[V\right]=\overset{D}{\underset{i=1}{\sum }}E\left[V_{i}\right]=D{\left(1-\frac{S}{D}\right)}^{R}. \end{array}$$

Hence, the number of expected miRNAs reported at least once is

(12) $$\begin{array}{l} E\left[\left(D-V\right)\right]=D-E\left[V\right]. \end{array}$$

Substituting the above-mentioned values for the parameters, we obtain that

- the expected number of miRNAs reported at least once after 50 iterations is 183; and
- the number of new miRNAs added in a further iteration would be 1.

As the number of sorted miRNAs in our experiment is 39 (see the Supplementary Material), this difference suggests that 50 runs are enough to conclude that all the true positives are included in the sorted list and so false negatives are unlikely.

### 2.7. Cox Regression Analysis for Evaluating miRNAs

The second part of the proposed method is for the evaluation of selected miRNAs using Cox regression analysis. The primary objective of this stage is to assess the importance of the miRNAs selected in the first part of the method, and this is done using Cox regression analysis for survival in 10 different cancer types. Expression data of the selected miRNAs and the associated clinical data are used for the Cox regression analysis. In the clinical data, the vital status of each patient, indicating whether the patient is still alive or has died, and the number of days since the last followup are taken into account when performing the survival analysis. Based on the expression levels and the clinical data of the selected miRNAs in each cancer type, the Cox coefficient, hazard ratio, and *p*-value are computed. A higher value of the Cox coefficient signifies greater importance of that miRNA to the respective cancer type. Moreover, the up- and down-regulation of all the selected miRNAs are observed to understand their behavior with respect to that particular cancer type based on change in expression in tumor and normal samples.

### 2.8. Complexity Analysis

Let *D* be the number of features and *n* the number of samples in the input dataset. As the available approximations may considerably lower the overall complexity, we discuss the complexity of each building block separately. A single step of SNE requires computing relations between all data points. We embed a transposed dataset, making an optimization step in *O*(*D*^2^) time. Computing SNE usually involves performing principal component analysis for preliminary dimension reduction, though its cost is negligible. The internal complexity of CMA-ES is estimated as *O*(*D*^2^), due to sampling and updating of the covariance matrix. The matrix needs to be factorized, which can be done by eigen decomposition in *O*(*D*^3^) time. Factorization does not happen in every generation, which gives *O*(*D*^2^) amortized time. Empirical evidence suggests that the sufficient number of objective function evaluations usually scales sub-quadratically with *D* (Ros and Hansen, 2008). The computation time is similar, since the vast majority of it is time spent training similar classifiers. On the other hand, the time complexity of Cox regression analysis is *O*(*nD*^2^) for a single run (Kelley, 1999).

## 3. Results and Discussion

The performance of the proposed method (SCES-FS) was tested on real miRNA expression datasets for 10 different cancer types and compared with the results of 16 existing methods, namely ESVM-RFE (Anaissi et al., 2016), LASSO (Tibshirani, 1996), NSGA-II-SE (Saha et al., 2017), MOGA (Mukhopadhyay and Maulik, 2013), SVM-nRFE (Peng et al., 2009), SVM-RFE (Guyon et al., 2002), CMIM (Fleuret, 2004), ICAP (Jakulin, 2005), SCAD (Fan and Li, 2001), JMI (Bennasar et al., 2015), CIFE (Brown et al., 2012), mRMR (Peng et al., 2005), FSCOX (Kim et al., 2014), DISR (Brown et al., 2012), SNRs (Mishra and Sahu, 2011), and RankSum (Troyanskaya et al., 2002) (see section 1 for the full names of these methods), as well as the results with all features (i.e., without feature selection).

### 3.1. Dataset Preparation and Parameter Setting

The miRNA expression and clinical datasets of bladder, breast, colon, glioblastoma, head and neck squamous cell, kidney renal clear cell, lung adenocarcinoma, lung squamous cell, ovarian, and uterine corpus endometrial carcinoma were obtained from TCGA. These 10 cancer types have also been studied previously (see, e.g., Jacobsen et al., 2013). Moreover, Hoadley et al. (2018) found that the characteristics of certain cancers out of 33 types provided in TCGA are overlapping in nature. As a result, 10–15 distinct groups of cancer were reported in Hoadley et al. (2018), which are similar to the cancer types studied in the present article. Our choice of cancer types was based on: (1) careful review of the literature, (2) the availability of tissue-specific tumor and normal samples to avoid the class imbalance problem in classification, and (3) the availability of common miRNA expression data for different cancer types and their corresponding clinical data. Thus, 10 cancer types were selected for our study. The expression data were generated using an Illumina high-throughput sequencing machine in the form of read counts of 199 miRNAs, normalized to reads per million, while the clinical data contain *gender, age, days since last followup*, and *vital status*. After removing miRNAs that contain more than 60% zeros in each cancer type and taking the miRNAs common to all the cancer types, we found 199 miRNAs and considered their expression in different cancer types. The number of samples and other details for each cancer type are given in Table 1; we also included 333 normal samples in the analysis with the expression of same miRNAs. For convenience, the expression datasets containing reads per million are further normalized onto a log_2_ scale. These processed datasets can be downloaded from the Supplementary Material website^3^. To construct the final ranking of miRNAs, the SCES-FS algorithm was run 50 times. Five-fold cross-validation was applied during each classification to avoid the issue of overfitting or underfitting. The parameters used in the experiments are shown in Table 2 and were obtained either experimentally or from the literature (Latinne et al., 2001; Oshiro et al., 2012; Hansen, 2016).

**Table 1:** Details of the data for 10 different cancer types.

**Cancer type**	**Code**	**No. of tumor samples**	**Gender**		**Average age**	**Average no. days from last followup**
			**Male**	**Female**		
Bladder urothelial carcinoma	BLCA	94	67	27	67.07	416.97
Breast invasive carcinoma	BRCA	255	0	255	58.28	1297.82
Colon adenocarcinoma	COAD	119	57	62	70.91	616.80
Glioblastoma multiforme	GBM	38	20	18	62.78	376.81
Head and neck squamous cell carcinoma	HNSC	298	218	80	60.98	1038.31
Kidney renal clear cell carcinoma	KIRC	146	91	55	59.99	1236.55
Lung adenocarcinoma	LUAD	61	29	32	65.62	743.09
Lung squamous cell carcinoma	LUSC	89	64	25	64.78	1296.87
Ovarian serous cystadenocarcinoma	OV	509	0	509	59.85	1020.87
Uterine corpus endometrial carcinoma	UCEC	98	0	98	62.32	1066.01

**Table 2:** Values of parameters.

**Symbol**	**Value**	**Description**
*C*	*I*	CMA-ES initial covariance matrix of size *H*
σ	0.3	Initial value of step-size control parameter
λ	200	Population size
μ	100	Number of parents
R	50	Number of runs
*N*	200	Maximum number of generations
*t*	0.5	Threshold for calculating subsets *S*_*i*_
α	0.5	Excessive attributes penalty term
Υ	10	Target number of miRNAs
γ	0.05	SVM RBF kernel parameter
C	1.0	SVM C constant
*K*	5	Value of *K* in *K*-NN
M	50	Number of trees in RF

### 3.2. Experimental Outcomes

The problem of finding miRNAs that can correctly distinguish different cancer types were posed as a multi-class classification task using expression data of miRNAs, and the importance of the selected miRNAs was evaluated using Cox regression analysis with the help of clinical data. The classification results of SCES-FS using the classifiers RF, SVM, NB, *K*-NN, and DT over 50 runs are reported in Table 3 and compared with the results of ESVM-RFE, LASSO, NSGA-II-SE, MOGA, SVM-nRFE, SVM-RFE, CMIM, ICAP, SCAD, JMI, CIFE, mRMR, FSCOX, DISR, SNR, and RankSum, as well as the results with all features (i.e., without feature selection). These results show that SCES-FS with RF achieved the highest classification accuracy, 96.881, with a standard deviation of 0.039, while the accuracy results of SCES-FS with SVM, NB, *K*-NN, and DT are 96.332 ± 0.194, 96.251 ± 0.168, 96.132 ± 0.369, and 94.232 ± 0.057, respectively, still better than those of the other existing feature selection methods.

**Table 3:** Number of features and classification accuracy of feature selection methods for five classifiers with five-fold cross-validation.

**Method**	**Number** **of features**	**RF**	**SVM**	**NB**	**K-NN**	**DT**
SCES-FS	17	96.881 ± 0.039	96.332 ± 0.194	96.251 ± 0.168	96.132 ± 0.369	94.232 ± 0.057
ESVM-RFE	22	95.684 ± 0.031	95.902 ± 0.193	92.672 ± 0.161	91.382 ± 0.369	90.429 ± 0.056
LASSO	48	95.601 ± 0.038	95.547 ± 0.191	92.582 ± 0.164	91.251 ± 0.367	90.241 ± 0.051
NSGA-II-SE	26	95.587 ± 0.033	95.537 ± 0.195	92.538 ± 0.166	91.183 ± 0.366	90.229 ± 0.052
MOGA	24	95.391 ± 0.036	95.531 ± 0.194	92.293 ± 0.161	90.338 ± 0.362	89.142 ± 0.055
SVM-nRFE	26	95.224 ± 0.032	95.321 ± 0.191	92.281 ± 0.167	90.106 ± 0.361	88.993 ± 0.051
SVM-RFE	28	95.048 ± 0.038	95.159 ± 0.199	92.116 ± 0.165	89.889 ± 0.366	88.691 ± 0.053
CMIM	28	94.299 ± 0.037	92.029 ± 0.193	90.683 ± 0.161	89.374 ± 0.369	89.161 ± 0.052
ICAP	27	93.721 ± 0.031	92.951 ± 0.192	90.874 ± 0.163	90.643 ± 0.362	87.057 ± 0.053
SCAD	25	91.972 ± 0.034	90.839 ± 0.194	90.003 ± 0.165	89.918 ± 0.366	87.495 ± 0.058
JMI	28	91.718 ± 0.031	90.602 ± 0.196	89.986 ± 0.166	88.639 ± 0.369	87.057 ± 0.051
CIFE	32	90.886 ± 0.034	89.072 ± 0.199	88.389 ± 0.162	87.261 ± 0.362	86.205 ± 0.056
mRMR	28	91.063 ± 0.032	89.753 ± 0.195	87.402 ± 0.161	87.254 ± 0.361	85.208 ± 0.059
FSCOX	23	89.298 ± 0.038	88.529 ± 0.198	87.505 ± 0.169	87.287 ± 0.368	85.498 ± 0.058
DISR	29	89.286 ± 0.031	88.276 ± 0.196	87.580 ± 0.167	87.321 ± 0.369	85.858 ± 0.053
SNR	30	87.866 ± 0.035	86.712 ± 0.193	85.749 ± 0.163	85.364 ± 0.365	84.042 ± 0.059
RankSum	32	86.633 ± 0.033	85.556 ± 0.199	85.466 ± 0.166	84.669 ± 0.366	84.322 ± 0.055
Without feature selection	199	86.428 ± 0.036	85.294 ± 0.191	85.183 ± 0.162	84.552 ± 0.367	84.118 ± 0.053

It is to be noted that our results have been verified with FSCOX, which computes important miRNAs via Cox regression on all miRNAs and subsequently uses them for the classification. It has been observed that the overall classification accuracy of FSCOX is less than the accuracy attained by SCES-FS as reported in Table 3. Other omics-based analyses, such as protein array, copy number variation (CNV), and methylation studies, also have potential applications in pan-cancer classification, as explained in Zhang et al. (2015, 2016, 2019), where the overall accuracy achieved in the range of 93–97%. The cancer dataset used in Zhang et al. (2015, 2016, 2019) was carefully selected for protein array, CNV, and methylation studies and is not directly suitable for experimenting with miRNAs, as it creates a class imbalance problem when selecting miRNA expression data for both tumor and normal cases. However, the overall accuracy of our method is higher than 96%, which is on the higher side of the accuracy range reported by Zhang et al. (2015, 2016, 2019), suggesting that our selected miRNAs can also be considered potential markers for pan-cancer classification.

The results of SCES-FS have been further validated by survival analysis, including Cox regression, network analysis, expression analysis using hierarchical clustering in the form of heatmaps, KEGG pathway analysis, GO enrichment analysis, and PPI network analysis, as described in the following.

#### 3.2.1. Survival Analysis

**Figure 2:**
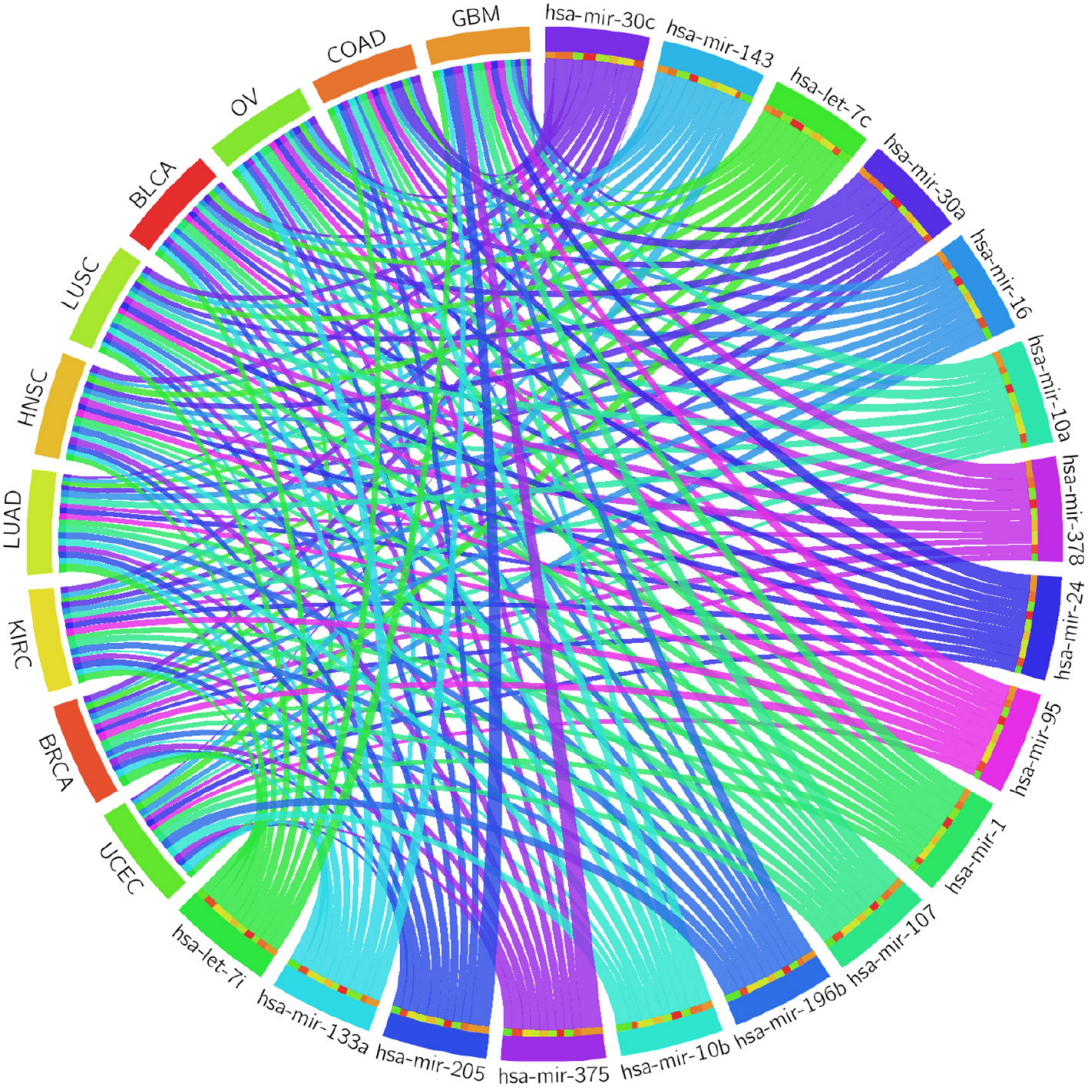
Circos plot of Cox regression analysis results: Cox coefficient values are used to graphically visualize the association of 17 miRNAs with ten cancer types; a broader band signifies a stronger association between the miRNA and the particular cancer type.

Table 4 reports the results of the Cox regression analysis, i.e., the Cox coefficient and hazard ratio values, for 10 cancer types in order to evaluate the importance of the 17 selected miRNAs with respect to these cancer types. The Cox regression analysis was performed by integrating the miRNA expression and clinical data to assess the effect of miRNA expression on cancer survival. Higher Cox coefficient and hazard ratio values indicate greater influence of the miRNA on the cancer type. For example, the miRNA hsa-mir-375 has the highest Cox coefficient, 0.9882, and hazard ratio, 1.7879, for the GBM cancer type. To help visualize the importance of each miRNA to the different cancer types, a circos plot of Table 4 is shown in Figure 2. In the figure, a broader band signifies a stronger association between the miRNA and the particular cancer type. Table 5 summarizes the cancer types that are most highly associated with the selected miRNAs. In this table, for each miRNA, the cancer type for which that miRNA has the highest Cox coefficient is shown, along with the associated *p*-value, false discovery rate (FDR), and up- or down-regulation of the miRNA expression in that cancer type. Similarly, Supplementary Table 2 gives the up- and down-regulations along with the corresponding FDR values of the selected miRNAs for all cancer types. The up- and down-regulations of the miRNAs are computed after evaluating the change in expression of the selected miRNAs between tumor samples and normal samples or the population for a particular cancer type using the ANOVA test; this is discussed further in section 3.2.3. Here, the change in expression is considered significant if the *p* < 0.05, and the up- or down-regulation is computed if the change in population expression is positive or negative, respectively. In summary, we find that hsa-mir-205, hsa-mir-10a, hsa-mir-196b, hsa-mir-10b, hsa-mir-375, hsa-mir-143, hsa-let-7c, hsa-mir-107, hsa-mir-378, hsa-mir-133a, hsa-mir-1, hsa-mir-30c, hsa-mir-16, hsa-mir-30a, hsa-let-7i, hsa-mir-24, and hsa-mir-95 are highly associated with GBM, UCEC, OV, BLCA, COAD, and BRCA.

**Table 4:** Results of Cox regression analysis of each selected miRNA.

**miRNA**	**BLCA**		**BRCA**		**COAD**		**GBM**		**HNSC**		**KIRC**		**LUAD**		**LUSC**		**OV**		**UCEC**	
	**Cox** **coefficient**	**Hazard** **ratio**	**Cox** **coefficient**	**Hazard** **ratio**	**Cox** **coefficient**	**Hazard** **ratio**	**Cox** **coefficient**	**Hazard** **ratio**	**Cox** **coefficient**	**Hazard** **ratio**	**Cox** **coefficient**	**Hazard** **ratio**	**Cox** **coefficient**	**Hazard** **ratio**	**Cox** **coefficient**	**Hazard** **ratio**	**Cox** **coefficient**	**Hazard** **ratio**	**Cox** **coefficient**	**Hazard** **ratio**
hsa-mir-205	−0.0344	0.9661	−0.0795	0.9234	0.1109	1.1172	0.9233	2.5177	0.0586	1.0600	0.0187	1.0189	0.1292	1.1379	−0.0449	0.9560	−0.0224	0.9778	−0.0039	0.9960
hsa-mir-10a	0.0618	1.0637	−0.0750	0.9277	−0.1644	0.8483	0.4881	1.6293	−0.0428	0.9580	−0.0122	0.9878	0.1193	1.1267	0.1619	1.1758	0.0569	1.0586	−0.4075	0.6652
hsa-mir-196b	−0.0333	0.9672	0.0089	1.0090	−0.2236	0.7996	0.2573	1.2935	−0.0095	0.9905	0.1367	1.1465	0.1124	1.1189	−0.0282	0.9721	0.0146	1.0147	0.4581	1.5811
hsa-mir-10b	0.0828	1.0864	−0.2213	0.8014	0.4042	1.4981	0.0795	1.0827	0.0597	1.0615	−0.2743	0.7600	0.1626	1.1766	0.0176	1.0178	−0.0218	0.9783	0.5080	1.6621
hsa-mir-375	−0.0226	0.9776	0.1334	1.1427	−0.0313	0.9691	0.9882	1.7879	0.0525	1.0539	−0.0051	0.9948	−0.0967	0.9078	−0.0340	0.9665	−0.0768	0.9260	0.0243	1.0246
hsa-mir-143	0.1158	1.1228	−0.2472	0.7809	−0.0759	0.9268	−0.0654	0.9366	−0.0630	0.9389	−0.0433	0.9575	−0.1486	0.8619	0.0053	1.0054	0.1504	1.1623	0.0770	1.0801
hsa-let-7c	0.1731	1.1890	−0.1825	0.8331	0.0702	1.0727	−0.1349	0.8737	0.0430	1.0440	−0.1158	0.8905	−0.1544	0.8569	0.0665	1.0687	0.0618	1.0637	0.0090	1.0090
hsa-mir-107	−0.0395	0.9611	0.1127	1.1193	0.0448	1.0459	0.5363	1.7096	−0.0389	0.9617	0.0405	1.0413	0.0885	1.0925	−0.0926	0.9114	−0.0919	0.9121	0.0609	1.0628
hsa-mir-378	−0.0286	0.9717	−0.1355	0.8732	0.1520	1.1641	0.5511	1.7352	0.0688	1.0712	−0.1881	0.8284	−0.1228	0.8843	0.1748	1.1910	0.0566	1.0583	−0.1596	0.8524
hsa-mir-133a	0.0610	1.0629	0.1604	1.1740	−0.1025	0.9025	0.2683	1.4481	−0.0393	0.9614	0.0328	1.0334	0.0810	1.0844	−0.0589	0.9427	0.4422	1.5561	0.2160	1.2411
hsa-mir-1	0.0630	1.0651	−0.1150	0.8913	−0.0637	0.9382	0.3702	1.3078	−0.0295	0.9708	−0.0208	0.9793	0.0089	1.0090	0.0954	1.1001	0.1580	1.1712	0.0084	1.0084
hsa-mir-30c	−0.0023	0.9976	−0.0550	0.9464	0.5053	1.6575	−0.0168	0.9833	0.0621	1.0641	−0.0121	0.9879	−0.4443	0.6412	0.1438	1.1547	−0.0056	0.9943	−0.6118	0.5423
hsa-mir-16	−0.0326	0.9678	0.0947	1.0993	−0.0634	0.9385	0.1409	1.1513	−0.0370	0.9636	0.0266	1.0270	0.0471	1.0482	−0.0732	0.9293	0.0058	1.0058	0.0447	1.0457
hsa-mir-30a	0.0845	1.0882	−0.1589	0.8530	0.8083	2.2441	0.0565	1.0582	0.0808	1.0841	−0.0779	0.9250	−0.1680	0.8452	0.0764	1.0794	0.0737	1.0765	−0.7061	0.4935
hsa-let-7i	0.1311	1.1401	0.4383	1.5501	0.1915	1.2110	0.2327	1.2621	−0.0354	0.9651	0.1394	1.1496	0.2574	1.2936	0.0702	1.0728	−0.1047	0.9005	0.0353	1.0360
hsa-mir-24	0.0191	1.0193	−0.0863	0.9172	0.0659	1.0681	0.4885	1.6298	0.0393	1.0401	−0.0345	0.9660	−0.0782	0.9246	0.0666	1.0688	0.0503	1.0516	−0.1175	0.8890
hsa-mir-95	−0.0345	0.9660	0.1185	1.1258	−0.0282	0.9721	0.2872	1.3326	0.0184	1.0186	0.1327	1.1419	0.0320	1.0325	0.0102	1.0102	0.0467	1.0478	−0.1247	0.8827

**Table 5:** Cancer type most strongly associated with each selected miRNA, based on Cox coefficient.

**miRNA**	**Cox** **coefficient**	**Hazard** **ratio**	**Cancer** **type**	***p*-value**	**FDR**	**Regulation** **(up ↑/down ↓)**	**PubMed** **ID**
hsa-mir-205	0.9233	2.5177	GBM	5.14E-03	5.46E-03	↓	23054677
hsa-mir-10a	0.4881	1.6293	GBM	6.17E-24	1.87E-23	↓	20444541
hsa-mir-196b	0.4581	1.5811	UCEC	2.84E-48	1.21E-47	↑	–
hsa-mir-10b	0.5080	1.6621	UCEC	4.71E-03	4.71E-03	↓	–
hsa-mir-375	0.9882	1.7879	GBM	1.80E-14	2.36E-14	↓	29110584
hsa-mir-143	0.1504	1.1623	OV	3.38E-57	1.79E-56	↓	25304686
hsa-let-7c	0.1731	1.1890	BLCA	3.85E-38	1.31E-37	↓	21464941
hsa-mir-107	0.5363	1.7096	GBM	5.42E-24	1.87E-23	↑	24213470
hsa-mir-378	0.5511	1.7352	GBM	5.04E-20	7.79E-20	↓	29088758
hsa-mir-133a	0.4422	1.5561	OV	2.78E-20	4.73E-20	↑	24944666
hsa-mir-1	0.3702	1.3078	GBM	9.18E-08	1.04E-07	↑	–
hsa-mir-30c	0.5053	1.6575	COAD	4.48E-17	6.34E-17	↓	–
hsa-mir-16	0.1409	1.1513	GBM	5.42E-24	1.87E-23	↑	25864039
hsa-mir-30a	0.8083	2.2441	COAD	2.88E-56	9.80E-56	↓	22287560
hsa-let-7i	0.4383	1.5501	BRCA	1.33E-43	2.83E-43	↑	26378051
hsa-mir-24	0.4885	1.6298	GBM	5.42E-24	1.87E-23	↓	25864039
hsa-mir-95	0.2872	1.3326	GBM	6.59E-24	1.87E-23	↑	28155650

#### 3.2.2. Network Analysis

**Figure 3:**
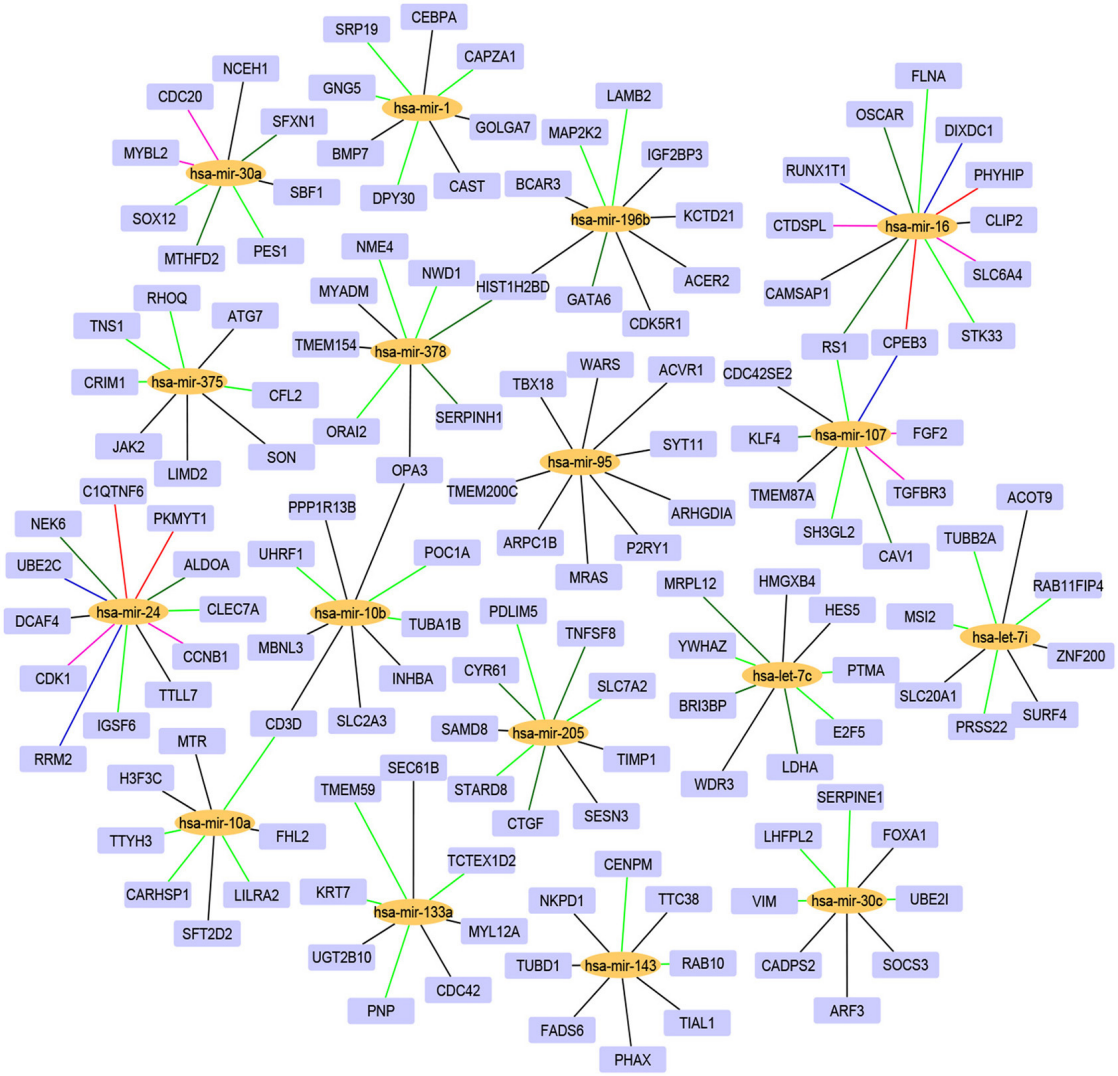
The interaction network of miRNAs and their target genes, where yellow nodes represent miRNAs and purple nodes represent genes. The edge colors red, blue, pink, dark green, light green, and black indicate that the corresponding miRNA-gene pair is associated with 6, 5, 4, 3, 2, and 1 cancer types, respectively.

For the 17 selected miRNAs, miRTarBase (Huang et al., 2020) was used to find their targets in order to elucidate their role in the different cancer types. To identify the most correlated targets, we computed the Pearson correlation between the expression values of miRNAs and mRNAs obtained from TCGA for the 10 cancer types and took the negative correlation value used in Zhou et al. (2015) as indicating strong association. The top five negatively correlated mRNAs associated with each of the 17 miRNAs are reported in Table 6; the rest are reported in Supplementary Material. To construct the interaction network, the miRNAs and their targets were ranked based on the cumulative negative correlation score and their presence in different cancer types as indicated by the association number. These results are reported in Supplementary Table 3. For example, hsa-mir-16 and its target, PHYHIP, are related to six cancer types and the cumulative negative correlation score is −4.142. Similarly, hsa-mir-24 is correlated with C1QTNF6 in another six cancer types, with a cumulative negative correlation score of −3.906. The subset of such targeted mRNAs is used to construct the interaction network shown in Figure 3. The network reveals that the miRNAs hsa-mir-205, hsa-mir-10a, hsa-mir-107, hsa-mir-378, and hsa-mir-16 and their targets {CYR61, STARD8, TNFSF8}, {TTYH3, CARHSP1, LILRA2}, {CPEB3, TGFBR3, FGF2}, {NME4, NWD1, ORAI2}, and {PHYHIP, CPEB3, CTDSPL} play a crucial role in different types of cancer. The red, blue, pink, dark green, light green, and black edges in Figure 3 signify that the number of cancer types associated with the corresponding pair of miRNA and target mRNA is 6, 5, 4, 3, 2, and 1, respectively. The targets of the miRNAs are investigated further using KEGG pathway, GO enrichment, and PPI network analysis in the following subsections in order to see their impact on the different types of cancer.

**Table 6:** Association of the 17 selected miRNAs and their top five targets in 10 cancer types.

	**BLCA**		**BRCA**		**COAD**		**GBM**		**HNSC**		**KIRC**		**LUAD**		**LUSC**		**OV**		**UCEC**	
		**Corr**.		**Corr**.		**Corr**.		**Corr**.		**Corr**.		**Corr**.		**Corr**.		**Corr**.		**Corr**.		**Corr**.
**miRNA**	**mRNA**	**score**	**mRNA**	**score**	**mRNA**	**score**	**mRNA**	**score**	**mRNA**	**score**	**mRNA**	**score**	**mRNA**	**score**	**mRNA**	**score**	**mRNA**	**score**	**mRNA**	**score**
	ZEB2	−7.45	E2F1	−3.29	SLC7A2	−9.95	PDLIM5	−5.28	RCAN2	−4.89	MAF	−4.68	ANGPTL7	−4.15	TRPV2	−6.43	PARD6B	−2.54	SLC7A2	−5.22
	ZEB1	−7.05	SATB2	−2.94	TIMP1	−9.63	ZNF707	−4.61	STARD8	−4.61	VEGFA	−4.58	ITM2A	−3.88	ALPK3	−6.20	PLCXD2	−2.10	SRC	−3.77
hsa-mir-205	SYT11	−7.02	RAB11FIP3	−2.88	SESN3	−9.43	ANKRD50	−4.25	ESRRG	−4.60	SLC37A4	−3.83	CPEB3	−3.54	STARD8	−6.16	C11orf74	−2.08	PARD6B	−3.55
	RCAN2	−6.99	ZFHX3	−2.70	SAMD8	−8.99	ERBB3	−4.25	ZEB1	−4.55	FLCN	−3.79	FAM19A1	−3.47	ENPP4	−6.04	FGF2	−2.05	PISD	−3.23
	LRRK2	−6.99	CDK1	−2.63	HOXA11	−8.78	YES1	−4.14	CTGF	−4.40	FGFR1OP	−3.55	CTGF	−3.17	LPCAT1	−6.02	SLC39A14	−1.97	BCL9L	−3.13
	NACC2	−5.33	E2F1	−3.93	H3F3C	−9.62	SLC2A3	−5.45	PANX1	−3.16	TTYH3	−6.29	TAF1D	−3.10	YOD1	−4.23	ARSK	−2.11	IRGQ	−5.32
	CLIC4	−5.29	BIRC5	−3.76	FHL2	−9.11	KIAA1143	−5.41	CARHSP1	−3.02	SCD	−6.28	DVL1	−2.93	ANP32E	−3.46	RIOK2	−2.04	FEM1A	−4.80
hsa-mir-10a	COL6A2	−5.04	PPM1G	−3.69	MTR	−8.99	YOD1	−5.36	FHL2	−2.79	CD3D	−6.19	AHCYL2	−2.85	CHMP1B	−3.37	ZBTB10	−2.03	E2F1	−4.78
	RAP1A	−4.79	TIMM50	−3.60	SFT2D2	−8.82	BCL6	−5.18	TFAP2A	−2.67	KLHL6	−6.12	PABPC1	−2.77	HNRNPF	−3.20	CHL1	−2.01	NF2	−4.77
	TGFB3	−4.77	TPI1	−3.54	NF2	−8.79	DUSP3	−5.12	EBNA1BP2	−2.44	COL6A2	−6.03	YAP1	−2.65	NOP16	−3.13	TRA2B	−1.99	CHRNA5	−4.71
	GATA6	−5.61	REEP5	−2.67	KCTD21	−9.89	MAP2K2	−5.92	PRUNE2	−3.57	HSD17B10	−2.28	MEIS1	−3.96	TGFBR2	−7.15	MYC	−2.45	HOXB7	−3.46
	PRUNE2	−5.26	DCTN4	−2.18	ACER2	−9.74	MYC	−4.55	BEST3	−3.05	CALM1	−2.16	TBRG1	−3.85	LAMB2	−5.98	MARS2	−1.97	HOXB8	−3.17
hsa-mir-196b	TGFBR2	−4.74	PBX1	−2.03	BCAR3	−9.68	PRKACA	−4.42	IGDCC4	−2.82	PBX1	−1.86	REEP5	−3.80	TRPC3	−5.67	GATA6	−1.91	SLC23A2	−2.95
	NR4A3	−4.65	SUOX	−1.81	IGF2BP3	−9.43	IARS	−4.11	KLHDC8B	−2.59	C14orf37	−1.43	TRPC3	−3.53	NR4A3	−5.52	ALDOA	−1.78	TGFBR3	−2.88
	SNX9	−4.59	TLE3	−1.78	HIST1H2BD	−9.10	HMGA1	−4.07	NR4A3	−2.33	SUOX	−1.41	TGFBR2	−3.32	GATA6	−5.11	GGA3	−1.63	GATA6	−2.61
	TPM1	−4.58	PLK1	−7.27	INHBA	−9.91	CMPK1	−6.23	RNF2	−2.50	TUBA1B	−5.41	MARVELD3	−3.34	LILRA2	−4.68	SDC1	−2.79	ASCL2	−3.78
	SFRP1	−4.13	BUB1	−6.83	MBNL3	−9.82	PDK3	−4.45	TTYH3	−2.43	HTATIP2	−5.11	NPEPPS	−2.92	AHCYL2	−4.52	INHBA	−2.72	GLB1L3	−3.53
hsa-mir-10b	MBNL1	−4.08	CCNA2	−6.76	OPA3	−9.61	EXOSC2	−4.29	UBE2Z	−2.20	LILRB2	−4.95	TRIM2	−2.87	S1PR2	−4.17	CMPK1	−2.69	PLA2G2C	−2.95
	PPP3CB	−3.86	MELK	−6.76	SLC2A3	−9.55	HNRNPF	−4.28	SLC5A5	−2.15	LILRA2	−4.86	FAHD1	−2.59	PAG1	−3.95	TMED5	−2.26	GPCPD1	−2.94
	SGCD	−3.78	POC1A	−6.70	PPP1R13B	−9.35	EIF1	−4.21	FZD2	−2.09	PLK1	−4.86	ALKBH4	−2.57	FGD4	−3.74	SLC2A3	−2.14	MSTO1	−2.73
	CTGF	−4.17	FAM89A	−6.15	SON	−9.94	REEP3	−5.94	CALU	−4.71	HEY1	−3.62	JAG1	−5.77	PIK3CA	−4.28	DPYSL3	−2.98	KLHDC8B	−4.97
	FSTL3	−4.09	RHOQ	−5.97	LIMD2	−9.81	BAK1	−5.02	COL12A1	−4.50	ZNF785	−3.38	KLF4	−5.58	NUP54	−3.67	CLDN1	−2.50	ASAP2	−4.73
hsa-mir-375	TNS1	−4.09	CFL2	−5.87	ATG7	−9.80	SPRED1	−4.97	EXT1	−4.28	CDCA7L	−3.28	MBD2	−5.43	ARNTL2	−3.66	IL1RAP	−2.06	SFT2D2	−4.48
	SH3D19	−4.01	ACSL4	−5.86	JAK2	−9.58	CARD8	−4.64	CCDC88A	−4.12	CARD8	−3.15	AKAP7	−5.21	JAG1	−3.58	COL12A1	−2.03	CLDN1	−4.22
	SAMD4A	−3.99	CELF2	−5.71	ESPNL	−9.54	ZNF799	−4.58	NETO2	−4.04	NETO2	−3.13	CRIM1	−5.14	USP46	−3.46	SEC23A	−1.98	TSC22D2	−4.20
	OTUB1	−5.34	CENPM	−5.21	NKPD1	−9.87	HIST1H2BG	−4.67	SDC1	−2.79	FSD2	−2.98	TIMM8A	−5.13	COX6B1	−4.67	MAT2A	−2.64	FHIT	−3.62
	CAPZA1	−4.88	PIK3R2	−4.73	TIAL1	−9.81	ANG	−4.30	RAB22A	−2.43	DTNB	−2.81	RAB10	−5.01	TRUB2	−4.34	SLC25A33	−2.35	SNX22	−2.89
hsa-mir-143	SYNPO2L	−4.88	STXBP2	−4.69	TUBD1	−9.51	RER1	−3.97	TMEM40	−2.39	TMEM120B	−2.48	PRMT3	−5.00	RPS19	−4.06	RACGAP1	−2.26	C4orf19	−2.85
	STXBP2	−4.79	LMNB2	−4.68	PHAX	−9.48	GLB1L	−3.90	RAB10	−2.38	MRPS25	−2.47	PTCD3	−4.93	AKT2	−4.01	GPSM2	−2.22	RDH10	−2.39
	KCNA7	−4.65	AP1S1	−4.66	TTC38	−9.44	ADCY2	−3.88	NKPD1	−2.30	QPRT	−2.40	C15orf48	−4.89	OTUB1	−3.94	CAPZA1	−2.17	ZNF117	−2.32
	YWHAZ	−4.49	CCNF	−5.94	HMGXB4	−9.81	TRIB1	−6.35	ITGA3	−5.38	RRM2	−4.23	LDHA	−7.24	COX6B1	−3.43	CASP3	−2.68	TNFRSF10B	−3.26
	SLC20A1	−4.16	CKS2	−5.89	HES5	−9.80	ACTB	−6.14	LDHA	−5.05	DLX4	−4.04	EZH2	−6.88	NAA20	−3.32	E2F6	−2.44	SOD2	−3.18
hsa-let-7c	EFHD2	−3.93	CCNB2	−5.84	YWHAZ	−9.79	THBS1	−6.11	MT2A	−4.81	TNFSF9	−3.96	HMGA1	−6.79	SF3B4	−3.17	COIL	−2.35	RNF7	−2.95
	MRPL12	−3.86	RRM2	−5.72	WDR3	−9.67	SLC20A1	−5.80	HMGA2	−4.77	CCNB2	−3.96	EIF4A3	−6.74	KIAA0391	−3.14	RNFT1	−2.27	CEBPB	−2.64
	PCGF3	−3.73	H2AFZ	−5.65	LYN	−9.47	FHL2	−5.54	PSMB2	−4.28	FANCI	−3.79	MRPL12	−6.67	YWHAZ	−3.11	GABPB1	−2.24	ICOSLG	−2.49
	GLP2R	−7.83	CAV1	−8.34	SMARCA5	−9.64	REL	−4.65	CPEB3	−5.23	FOXC1	−8.31	RS1	−8.88	RS1	−8.75	SUN2	−4.14	CPEB1	−5.76
	PER1	−7.67	FGF2	−8.05	TMEM87A	−9.63	PIK3R1	−3.59	CHRM1	−4.70	PLAG1	−8.24	SH3GL2	−8.86	ALDH3B1	−6.89	ERN1	−2.97	CYSLTR2	−3.71
hsa-mir-107	CPEB1	−7.62	FOXO1	−7.82	CDC42SE2	−9.52	ZBTB38	−3.43	AMOT	−4.09	CPEB3	−8.13	TGFBR3	−8.45	LATS2	−6.77	VCAN	−2.96	FGF2	−3.41
	NFIA	−7.32	DST	−7.71	PURA	−9.42	CAV1	−3.36	TGFBR3	−3.51	SH3GL2	−8.09	CAV1	−8.26	PRKCE	−6.36	PAG1	−2.85	SLC28A1	−2.91
	DMPK	−7.00	KLF4	−7.62	UBE2Q1	−9.04	ADORA3	−3.08	NFIA	−3.36	CKMT1A	−7.93	FGF2	−8.15	PAG1	−6.34	YWHAH	−2.61	DAPK1	−2.73
	ENO1	−5.57	PTBP1	−6.96	TMEM154	−9.76	NWD1	−6.56	SERPINH1	−6.16	PRKD2	−5.07	BYSL	−6.95	MELK	−4.10	KLHL7	−3.51	MYOZ3	−3.84
	TTC4	−5.48	RABEP2	−6.75	MYADM	−9.69	RTN3	−6.27	ENAH	−5.83	PML	−4.96	ALDOA	−6.91	PRMT1	−3.81	IGDCC3	−3.48	IGSF3	−3.45
hsa-mir-378	MRPL37	−5.37	BBC3	−6.68	OPA3	−9.57	CYP2U1	−5.99	MARVELD1	−5.48	LDHA	−4.71	LDHA	−6.78	PTOV1	−3.50	ENAH	−3.25	NLGN2	−3.35
	CDK4	−5.27	HIST1H2BD	−6.65	UGT8	−9.55	WDR5B	−5.63	FBLIM1	−5.46	ORAI2	−4.67	P4HB	−6.71	ENO1	−3.27	IGSF3	−3.19	MSC	−3.27
	FEN1	−5.08	DCTPP1	−6.34	KCNN1	−9.54	ENPP4	−5.50	MYO1B	−5.39	STOML1	−4.50	PAICS	−6.70	DCTPP1	−3.19	VAMP4	−3.16	YPEL1	−3.26
	IGF2BP1	−3.91	MEG3	−5.74	TMEM59	−9.63	NR3C1	−4.30	PIGR	−2.49	PRDM16	−7.24	FERMT2	−3.94	PRDM16	−3.79	VEGFA	−2.21	PIGR	−2.42
	DEK	−3.52	PIGR	−5.39	UGT2B10	−9.30	ANGEL2	−4.28	PIAS2	−2.40	CMTM4	−6.94	ANGPT4	−3.84	MLEC	−3.72	AFTPH	−1.90	BCL3	−2.28
hsa-mir-133a	CDK5R1	−3.49	PER2	−5.30	CDC42	−9.08	FAM160B1	−3.53	RBMXL1	−2.28	KCNQ1	−6.81	ZEB1	−3.75	ANGPT4	−3.53	CIAO1	−1.79	MYPN	−2.19
	SUPT16H	−3.43	NGFR	−4.77	SEC61B	−9.03	MMP14	−3.21	CIAO1	−2.23	ERBB2	−6.77	GRID1	−3.45	ARHGAP31	−3.50	KRT7	−1.72	KCNQ1	−2.08
	TCTEX1D2	−3.37	NR3C1	−4.65	MYL12A	−8.03	UBA2	−3.20	AFTPH	−2.23	PNP	−6.67	ZFP28	−3.30	ZEB1	−3.41	NR2C2	−1.64	NFAM1	−1.83
	NCAPG	−6.57	PTBP1	−5.38	BMP7	−9.88	SLC8A1	−5.20	LHX4	−3.32	VEGFA	−5.09	RFC5	−5.15	EFTUD2	−4.76	IFI44	−2.33	CD63	−2.85
	PTBP1	−6.33	ATP13A1	−4.69	CAST	−9.73	C1orf27	−5.03	FANCI	−3.32	ANGPTL4	−4.62	KIF4A	−4.92	IQGAP3	−4.72	MYEF2	−1.98	PPIB	−2.81
hsa-mir-1	RCC2	−6.23	OCIAD2	−4.54	CEBPA	−9.72	SCYL3	−4.94	SFXN1	−3.29	NETO2	−4.58	MAD2L1	−4.88	DSG2	−3.90	TWF1	−1.91	MTHFS	−2.72
	UHRF1	−6.21	KIAA1522	−4.54	GOLGA7	−9.67	LZTFL1	−4.80	BRI3BP	−3.27	KAT2A	−4.53	MTHFD2	−4.83	EIF4G1	−3.80	FOLR1	−1.90	MACROD1	−2.44
	SFXN1	−6.17	RCC2	−4.51	OAT	−9.63	WDR11	−4.78	NCAPD3	−3.01	HPS4	−4.44	SPC24	−4.76	SFXN1	−3.72	YTHDF2	−1.86	CRELD2	−2.24
	PAM	−4.93	NRBP1	−3.56	SOCS3	−9.78	MARCKSL1	−5.82	SNAI2	−3.65	VIM	−7.03	TOMM5	−4.76	BIRC5	−4.04	ETS1	−3.27	PSMD7	−4.26
	CXCL11	−4.92	RUNX2	−3.11	ARF3	−9.49	VASH1	−5.63	SERPINE1	−3.30	LHFPL2	−6.51	RPS3	−4.70	MYBL2	−4.03	MTDH	−3.15	UBE2I	−4.23
hsa-mir-30c	SNAI2	−4.22	PRDM1	−2.94	FOXA1	−9.48	ETS1	−5.40	SLC7A5	−3.22	SH3GL1	−6.40	MRPS16	−4.43	SLC7A5	−3.91	ITGB3	−3.15	NDUFA12	−3.64
	ADAM9	−3.84	CASP3	−2.92	UBE2I	−9.40	DLL4	−5.37	CTSC	−3.04	IFNAR2	−6.33	BIRC5	−4.33	ECT2	−3.85	RRM2	−2.99	LLPH	−3.63
	MGAT2	−3.79	PHTF2	−2.82	CADPS2	−8.43	SOX12	−4.96	SLC38A7	−3.03	BIRC5	−6.22	UXT	−4.29	RRM2	−3.81	CASP3	−2.99	PPP2R1B	−3.58
	PHYHIP	−8.38	DMD	−8.17	ZYX	−9.96	HGF	−5.72	CPEB3	−5.13	DMRT2	−9.17	RS1	−8.88	DLC1	−9.04	SPRYD3	−2.93	PTPRT	−6.30
	NEGR1	−8.11	GNAL	−8.17	CLIP2	−9.96	CCPG1	−5.68	TMEM100	−4.95	C1orf226	−8.94	TGFBR3	−8.70	RS1	−8.82	ZCCHC3	−2.37	SLC6A4	−6.24
hsa-mir-16	GLP2R	−8.01	PLSCR4	−8.15	CAMSAP1	−9.95	IRAK3	−5.49	PDCD4	−4.56	SLC9A2	−8.89	WNT3A	−8.65	NEGR1	−8.32	CCND1	−2.22	PHYHIP	−5.34
	GNAL	−7.85	RBMS3	−8.10	TLL1	−9.93	PDXK	−5.45	PDK4	−4.51	SPTBN2	−8.87	SLC6A4	−8.54	SLC6A4	−8.09	BTRC	−2.19	PLSCR4	−5.18
	DIXDC1	−7.73	CDC14B	−7.83	KATNAL1	−9.90	PHYHIP	−5.42	ZBTB16	−4.30	SLC6A4	−8.84	AGER	−8.53	KDR	−7.97	BAMBI	−2.11	KCND3	−4.74
	BAZ1B	−4.25	CBX2	−4.83	NCEH1	−9.64	PIK3R2	−5.50	FSCN1	−5.26	SERPINE1	−6.55	SFXN1	−6.36	KIF11	−6.08	C8orf76	−2.18	PPP2R1B	−4.41
	LMNB1	−4.22	CCNE2	−4.32	SBF1	−9.61	PES1	−5.43	FBXO45	−5.07	TGFBI	−6.26	PHTF2	−6.34	KPNA2	−5.87	MTHFD2	−2.07	GRPEL2	−4.39
hsa-mir-30a	MYBL2	−4.16	RRM2	−4.23	SFXN1	−9.31	SIX4	−5.41	YWHAZ	−4.77	RUNX2	−6.24	PAICS	−6.24	CDC20	−5.84	CASP3	−2.01	DCTN4	−4.16
	RRM2	−4.10	C8orf76	−4.19	SOX12	−9.21	THOC5	−5.34	SLC16A1	−4.62	ITGA5	−6.15	TUBB3	−6.16	RRM2	−5.70	QRFPR	−1.96	IDH1	−4.11
	PAICS	−3.98	MYBL2	−4.10	RTP4	−9.16	NR2F6	−5.16	TUBB3	−4.57	CARS	−6.08	NUPL2	−6.14	PAICS	−5.56	CBX3	−1.75	STMN1	−4.06
	SYNJ2BP	−4.59	CRY2	−3.32	SLC20A1	−9.29	ONECUT2	−3.74	SDR42E1	−1.81	ANKRD46	−7.49	QDPR	−4.07	USP47	−3.18	HMGA2	−3.31	MEIS3P1	−3.39
	MYOCD	−4.31	MTUS1	−2.79	MSI2	−9.25	RABL2A	−3.70	CD59	−1.67	PAFAH2	−7.28	AHR	−3.23	GGA3	−3.10	ZCCHC3	−3.09	PBX2	−3.23
hsa-let-7i	ACTA1	−4.30	DUSP1	−2.64	TUBB2A	−9.14	CDV3	−3.69	PRSS22	−1.46	TGFBR3	−6.99	ASCL1	−3.22	PIK3C2A	−3.05	COPS8	−2.98	SEMA4C	−2.55
	SEMA4C	−4.08	DNAH9	−2.53	SURF4	−8.86	NCKIPSD	−3.61	GLO1	−1.40	DNAJC28	−6.96	DNAH9	−3.17	TSC22D2	−2.93	PBX2	−2.59	RNF144B	−2.53
	KCNB1	−3.96	DNAJC28	−2.31	ACOT9	−8.67	C5orf51	−3.55	GRPEL2	−1.17	NAT8L	−6.96	TGFBR3	−3.02	IGF2BP1	−2.91	HMGA1	−2.57	DDOST	−2.51
	MEN1	−6.99	PKMYT1	−7.61	TTLL7	−9.87	FLCN	−5.28	TGFB1	−6.65	NETO2	−8.35	CCNB1	−7.43	AURKB	−7.58	KAZALD1	−2.84	PRSS8	−2.95
	CDK1	−6.61	TRIM11	−7.45	DCAF4	−9.79	CYP20A1	−5.18	C1QTNF6	−6.46	STX4	−8.13	ALDOA	−7.41	UBE2C	−7.57	PLAGL2	−2.66	AGPAT2	−2.87
hsa-mir-24	ADPGK	−6.48	CCNB1	−7.28	CCL4	−9.73	ADPGK	−4.28	MMP14	−6.16	C1QTNF6	−8.08	RRM2	−7.39	CCNB1	−7.50	BTBD3	−2.44	PKMYT1	−2.67
	UBE2C	−6.45	UBE2C	−7.27	SIT1	−9.69	SMYD4	−4.27	FSCN1	−5.97	EHD2	−8.06	IMP4	−7.28	CCNA2	−7.29	ZNF107	−2.30	TNIP2	−2.62
	TRIM11	−6.44	CDK1	−7.23	OLR1	−9.63	MDM4	−4.18	NETO2	−5.94	CDKN2A	−8.03	LDHA	−7.25	RRM2	−7.04	DNAJC10	−2.23	ATL3	−2.58
	DGKB	−4.54	ITM2B	−3.83	TBX18	−9.74	CREBL2	−4.81	SCD	−2.30	RDH11	−3.52	FEM1C	−4.23	ZNF699	−3.27	ZNF460	−1.45	ITPRIPL2	−3.77
	ACTC1	−4.27	CREBL2	−3.52	MRAS	−8.52	MCTS1	−4.80	RDH11	−2.20	MOCS2	−3.41	ZBTB43	−4.15	PER1	−2.97	FEM1C	−1.36	CRK	−3.19
hsa-mir-95	FOXP2	−3.80	INTU	−3.50	ACVR1	−8.39	B3GNT2	−4.09	USP8	−2.07	METAP2	−3.16	ITM2B	−3.86	CDKN1A	−2.94	B3GNT2	−1.29	FAM126B	−3.06
	PTBP2	−3.79	ACVR1	−3.49	WARS	−8.15	TRIP4	−3.81	HFE	−1.98	ZNF711	−3.10	CREBL2	−3.84	REL	−2.87	DGKB	−1.26	FOXJ3	−3.01
	NXPH3	−3.65	SNX1	−3.35	ARPC1B	−8.10	CEBPD	−3.80	B3GNT2	−1.72	AVPI1	−3.05	CELF2	−3.79	DUSP18	−2.80	CACNG8	−1.26	LRRC58	−2.96

#### 3.2.3. Expression Analysis

**Figure 4:**
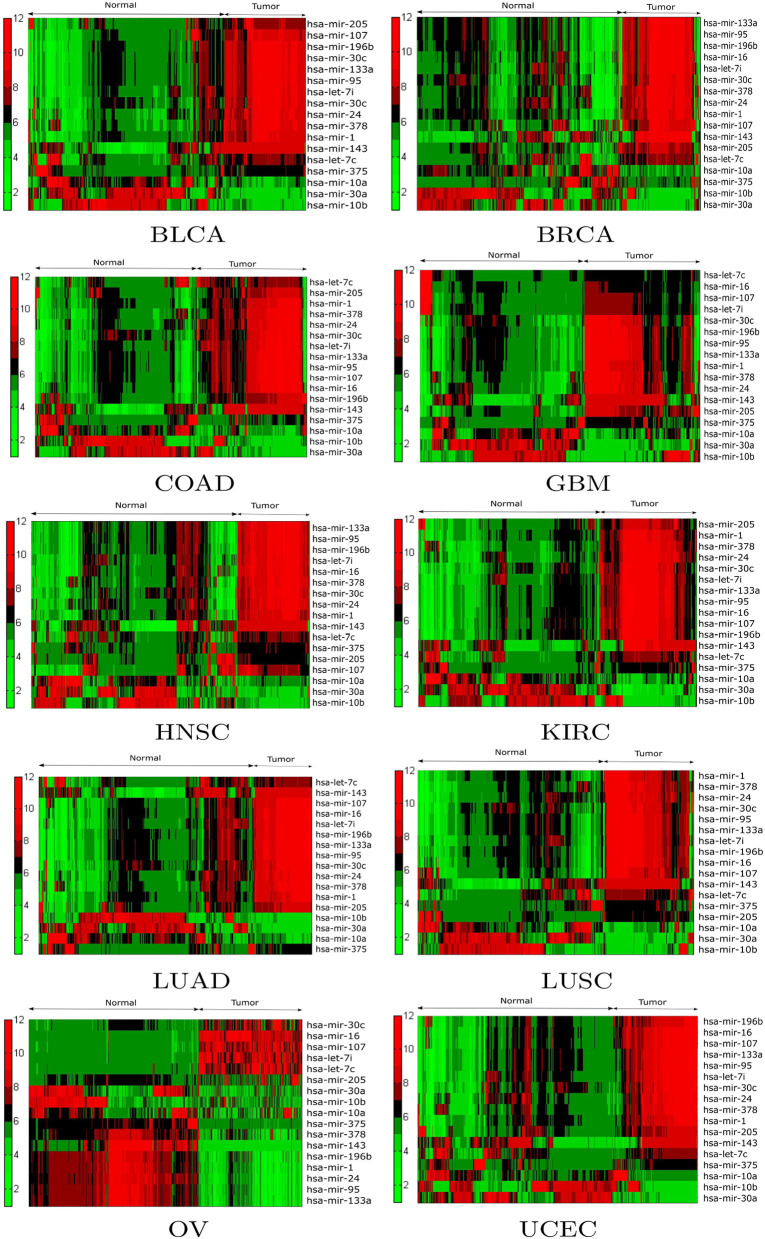
Hierarchical clustering results of the differentially expressed miRNAs for the BLCA, BRCA, COAD, GBM, HNSC, KIRC, LUAD, LUSC, OV, and UCEC datasets. Red indicates high expression levels, green low expression levels, and black not significantly expressed samples.

Expression analysis was conducted using a one-way ANOVA test in order to evaluate the statistical significance of the differential expression of the 17 selected miRNAs. Alternative techniques can also be used (Conesa et al., 2016; Costa-Silva et al., 2017; Crow et al., 2019). To perform the test, the population of patients was divided into tumor and normal groups for a given miRNA in a particular cancer type. As a result of ANOVA, significant (*p* < 0.05) changes in expression were observed between the tumor and normal groups for the 17 selected miRNAs. For example, the *p*-values of hsa-mir-10a, hsa-mir-196b, hsa-mir-10b, hsa-mir-375, hsa-mir-143, hsa-let-7c, hsa-mir-107, hsa-mir-378, hsa-mir-133a, and hsa-mir-30c were 6.17E-24, 2.84E-48, 4.71E-03, 1.80E-14, 3.38E-57, 3.85E-38, 5.42E-24, 5.04E-20, 2.78E-20, and 4.48E-17 respectively. Additionally, box plots of the selected miRNAs in tumor and normal samples are provided in Supplementary Figure 3. To investigate the relationship between the expression levels of miRNAs for each cancer type, hierarchical clustering was performed on the tumor and normal samples of the 17 selected miRNAs. The results are shown in Figure 4, from which the change in expression levels of the miRNAs between tumor and normal samples is evident. In the cluster plots, red indicates high expression levels and green low expression levels; black corresponds to not significantly expressed samples.

#### 3.2.4. KEGG Pathway Analysis

In order to perform KEGG pathway analysis for the 17 selected miRNAs in 10 cancer types, their targets were identified based on negative correlation values as described in section 3.2.2. Then, these target mRNAs were used in the DIANA tool (Vlachos et al., 2015) separately to identify significant KEGG pathways associated with the selected miRNAs in different cancer types. The five most significant pathways for each of the 17 miRNAs according to the FDR-corrected *p*-value within 5% statistical significance for the different cancer types are reported in Table 7. The detailed pathways of the 17 miRNAs with all their targets are presented in Supplementary Material. It can be seen from Table 7 that the most significantly enriched pathways are involved in various cancer types. For example, hsa-mir-205 and hsa-mir-133a are found to be enriched in pathways relating to *hsa05206: MicroRNAs in cancer* for nine cancer types. Similarly, hsa-mir-196b is found to be enriched in the pathway of *hsa05210: Colorectal cancer* for all 10 cancer types, with the FDR-corrected *p*-values of BLCA, BRCA, COAD, GBM, HNSC, KIRC, LUAD, LUSC, OV, and UCEC being 2.24E-07, 2.30E-06, 2.31E-07, 7.00E-04, 2.36E-07, 4.54E-06, 8.48E-05, 2.38E-06, 2.24E-07, and 2.20E-07, respectively. In addition, critical pathways such as the *PI3K-Akt signaling pathway, p53 signaling pathway, Bladder cancer, Pancreatic cancer, Prostrate cancer*, and *Lung cancer* are also found for the 17 miRNAs with FDR-corrected *p*-values within 5% statistical significance in 10 different cancer types. The presence of such critical pathways for the 17 selected miRNAs suggests that these miRNAs play a significant role in various cancer types, including the 10 types considered in this paper.

**Table 7:** Five significant KEGG pathways for each of the 17 selected miRNAs in 10 cancer types.

		**FDR-corrected** **p-value**									
**miRNA**	**Pathway**	**BLCA**	**BRCA**	**COAD**	**GBM**	**HNSC**	**KIRC**	**LUAD**	**LUSC**	**OV**	**UCEC**
	hsa05206: MicroRNAs in cancer	2.20E-03	–	1.60E-04	1.73E-05	2.23E-02	1.02E-06	2.20E-04	1.70E-04	2.00E-03	2.40E-03
	hsa05202: Transcriptional misregulation in cancer	1.33E-02	–	1.62E-02	–	–	1.11E-02	2.15E-02	–	9.20E-03	–
hsa-mir-205	hsa05219: Bladder cancer	–	1.06E-02	–	–	–	3.54E-02	–	–	–	6.30E-03
	hsa05205: Proteoglycans in cancer	–	–	–	2.38E-02	–	1.73E-02	–	–	–	6.30E-03
	hsa04520: Adherens junction	2.13E-02	–	–	–	–	–	2.80E-02	–	–	–
	hsa05016: Huntington's disease	1.27E-02	–	–	–	–	–	–	4.64E-02	–	–
	hsa04218: Cellular senescence	–	–	–	–	2.90E-03	3.01E-02	–	–	–	–
hsa-mir-10a	hsa04714: Thermogenesis	1.27E-02	–	–	–	–	–	–	–	–	–
	hsa04510: Focal adhesion	1.27E-02	–	–	–	–	–	–	–	–	–
	hsa04213: Longevity regulating pathway—multiple species	1.27E-02	–	–	–	–	–	–	–	–	–
	hsa05210: Colorectal cancer	2.24E-07	2.30E-06	2.31E-07	7.00E-04	2.36E-07	4.54E-06	8.48E-05	2.38E-06	2.24E-07	2.20E-07
	hsa01522: Endocrine resistance	2.77E-07	5.58E-07	–	–	2.92E-07	5.08E-06	8.58E-05	5.75E-07	–	–
hsa-mir-196b	hsa05215: Prostate cancer	2.39E-06	3.00E-06	–	–	2.51E-06	3.81E-05	–	3.09E-06	3.42E-06	–
	hsa05161: Hepatitis B	–	3.00E-06	–	1.52E-05	3.61E-07	–	8.58E-05	3.09E-06	5.15E-07	–
	hsa04915: Estrogen signaling pathway	2.15E-06	2.88E-06	–	–	2.26E-06	3.27E-05	–	2.97E-06	–	–
	hsa05169: Epstein-Barr virus infection	–	3.28E-02	–	2.40E-03	2.90E-02	1.10E-04	–	–	1.19E-02	–
	hsa04550: Signaling pathways regulating pluripotency of stem cells	–	–	1.50E-03	–	1.48E-02	–	–	–	1.19E-02	–
hsa-mir-10b	hsa05206: MicroRNAs in cancer	2.50E-03	–	–	–	–	–	–	–	1.19E-02	–
	hsa04110: Cell cycle	–	9.10E-03	–	–	–	2.80E-04	–	–	–	–
	hsa04914: Progesterone-mediated oocyte maturation	–	1.34E-02	–	–	4.95E-02	–	–	–	–	–
	hsa01521: EGFR tyrosine kinase inhibitor resistance	7.00E-04	1.09E-02	–	–	–	–	–	–	7.50E-04	2.80E-03
	hsa04550: Signaling pathways regulating pluripotency of stem cells	4.02E-02	1.09E-02	–	–	–	–	–	–	–	–
hsa-mir-375	hsa04066: HIF-1 signaling pathway	–	–	2.32E-02	–	–	–	–	4.60E-03	–	–
	hsa05165: Human papillomavirus infection	–	–	2.32E-02	8.70E-03	–	–	–	–	–	–
	hsa05224: Breast cancer	–	–	–	6.00E-03	–	1.90E-03	–	–	–	–
	hsa05230: Central carbon metabolism in cancer	–	1.00E-03	–	–	4.09E-02	–	–	–	–	–
	hsa05213: Endometrial cancer	–	1.00E-03	–	–	–	–	–	–	–	–
hsa-mir-143	hsa05206: MicroRNAs in cancer	–	1.00E-03	–	–	–	–	–	–	–	–
	hsa05205: Proteoglycans in cancer	–	1.00E-03	–	–	–	–	–	–	–	–
	hsa05161: Hepatitis B	–	1.00E-03	–	–	–	–	–	–	–	–
	hsa05206: MicroRNAs in cancer	–	4.05E-02	1.85E-02	2.20E-04	–	1.10E-04	–	–	–	–
	hsa04115: p53 signaling pathway	–	–	–	2.90E-03	–	1.63E-05	4.43E-02	–	–	–
hsa-let-7c	hsa04110: Cell cycle	–	6.10E-03	–	–	–	–	4.43E-02	–	–	–
	hsa05222: Small cell lung cancer	–	4.05E-02	–	–	–	4.10E-04	–	–	–	–
	hsa04215: Apoptosis—multiple species	–	4.05E-02	–	–	–	–	4.43E-02	–	–	–
	hsa05200: Pathways in cancer	2.50E-03	1.30E-03	–	1.31E-02	–	–	2.40E-03	4.70E-04	7.38E-05	–
	hsa01521: EGFR tyrosine kinase inhibitor resistance	5.50E-03	1.30E-03	8.20E-03	–	–	–	5.20E-03	5.60E-03	3.50E-04	–
hsa-mir-107	hsa05165: Human papillomavirus infection	8.60E-03	1.30E-03	1.41E-02	–	–	–	–	8.80E-03	2.30E-03	–
	hsa04151: PI3K-Akt signaling pathway	5.50E-03	1.40E-03	–	–	–	–	–	5.60E-03	2.70E-03	–
	hsa05224: Breast cancer	5.50E-03	–	–	–	–	–	5.30E-03	–	–	–
	hsa03013: RNA transport	1.49E-02	–	–	–	–	–	3.11E-02	2.04E-02	–	–
	hsa03010: Ribosome	–	–	–	–	–	–	6.60E-04	2.04E-02	–	–
hsa-mir-378	hsa01100: Metabolic pathways	–	–	–	–	–	–	2.56E-02	2.04E-02	–	–
	hsa00010: Glycolysis/Gluconeogenesis	–	–	–	–	–	–	2.56E-02	2.04E-02	–	–
	hsa05224: Breast cancer	–	–	–	–	1.25E-02	–	–	–	–	–
	hsa05206: MicroRNAs in cancer	7.80E-03	5.80E-03	1.76E-05	1.60E-04	4.70E-03	2.21E-02	1.92E-05	1.40E-06	7.20E-03	–
	hsa05215: Prostate cancer	7.80E-03	1.60E-04	–	3.90E-03	7.90E-04	2.21E-02	–	8.00E-04	7.20E-03	–
hsa-mir-133a	hsa05212: Pancreatic cancer	5.50E-04	–	4.20E-03	–	5.50E-04	2.21E-02	–	–	6.20E-03	–
	hsa01524: Platinum drug resistance	3.30E-03	–	2.71E-02	–	1.70E-03	–	–	–	6.20E-03	–
	hsa05205: Proteoglycans in cancer	–	–	–	3.10E-03	–	–	8.34E-05	8.00E-04	–	–
	hsa03030: DNA replication	1.34E-10	1.83E-07	–	–	6.25E-06	7.60E-03	6.10E-09	1.16E-05	–	–
	hsa03430: Mismatch repair	3.80E-04	7.10E-03	–	–	3.10E-04	–	4.00E-04	1.75E-02	–	–
hsa-mir-1	hsa04110: Cell cycle	2.50E-04	9.20E-03	–	–	2.10E-04	–	8.74E-08	–	–	–
	hsa03015: mRNA surveillance pathway	3.64E-02	–	–	–	–	–	–	1.01E-02	–	–
	hsa05166: HTLV-I infection	4.42E-02	–	–	–	–	–	–	–	–	–
	hsa05206: MicroRNAs in cancer	1.79E-05	–	2.90E-03	–	–	2.04E-05	–	–	1.21E-02	–
	hsa05200: Pathways in cancer	–	8.81E-05	–	2.40E-03	2.07E-02	3.40E-03	–	–	–	–
hsa-mir-30c	hsa05211: Renal cell carcinoma	–	2.30E-03	–	2.09E-02	6.40E-03	–	1.42E-02	–	–	–
	hsa04141: Protein processing in endoplasmic reticulum	–	2.30E-03	–	–	1.93E-02	–	1.04E-02	2.56E-02	–	–
	hsa04380: Osteoclast differentiation	2.71E-02	–	5.10E-03	–	–	–	–	–	9.20E-03	–
	hsa04510: Focal adhesion	1.90E-03	5.80E-03	–	–	–	–	–	–	–	–
	hsa04010: MAPK signaling pathway	1.31E-02	6.30E-03	–	–	–	–	–	–	–	–
hsa-mir-16	hsa05200: Pathways in cancer	1.58E-02	–	–	–	–	–	7.42E-05	–	–	–
	hsa04151: PI3K-Akt signaling pathway	–	2.60E-03	–	–	–	–	–	–	3.20E-03	–
	hsa05206: MicroRNAs in cancer	–	6.30E-03	–	–	–	–	7.40E-04	–	–	–
	hsa04110: Cell cycle	–	4.39E-06	–	3.90E-03	–	5.81E-05	2.30E-03	1.93E-06	–	–
	hsa05206: MicroRNAs in cancer	–	–	–	2.10E-04	2.28E-02	6.20E-04	–	5.70E-03	–	–
hsa-mir-30a	hsa05203: Viral carcinogenesis	–	5.50E-04	–	–	–	1.80E-03	–	–	2.14E-02	–
	hsa05130: Pathogenic *Escherichia coli* infection	–	1.24E-02	–	–	2.28E-02	–	–	5.70E-03	–	–
	hsa03013: RNA transport	–	–	–	–	–	–	2.00E-03	5.70E-03	–	–
	hsa05206: MicroRNAs in cancer	–	–	9.80E-03	–	1.97E-02	–	1.80E-04	–	–	–
	hsa04550: Signaling pathways regulating pluripotency of stem cells	1.20E-03	–	–	–	–	–	6.20E-04	–	–	–
hsa-let-7i	hsa04066: HIF-1 signaling pathway	1.20E-03	–	–	–	–	–	6.80E-04	–	–	–
	hsa05130: Pathogenic *Escherichia coli* infection	–	–	1.60E-03	–	1.97E-02	–	–	–	–	–
	hsa05225: Hepatocellular carcinoma	1.01E-02	–	–	–	–	–	–	–	–	–
	hsa04110: Cell cycle	1.29E-06	1.57E-07	–	–	9.85E-09	5.43E-05	4.19E-10	4.40E-10	–	–
	hsa03030: DNA replication	2.13E-07	3.59E-06	–	–	1.10E-04	–	1.20E-03	1.03E-07	–	–
hsa-mir-24	hsa04218: Cellular senescence	–	4.00E-03	–	–	1.10E-04	1.50E-04	9.90E-04	4.20E-04	–	–
	hsa04115: p53 signaling pathway	7.49E-05	1.30E-03	–	–	8.40E-04	–	1.20E-03	–	–	–
	hsa00240: Pyrimidine metabolism	4.50E-04	–	–	–	–	–	–	4.28E-05	–	–
	hsa05211: Renal cell carcinoma	4.20E-03	–	4.93E-02	–	–	–	4.30E-03	4.10E-03	–	–
	hsa05220: Chronic myeloid leukemia	–	–	4.93E-02	–	–	–	2.26E-02	–	–	–
hsa-mir-95	hsa05219: Bladder cancer	–	–	4.93E-02	–	–	–	4.90E-03	–	–	–
	hsa05206: MicroRNAs in cancer	–	–	4.93E-02	–	–	–	2.79E-02	–	–	–
	hsa05203: Viral carcinogenesis	–	–	4.93E-02	–	–	–	1.67E-02	–	–	–

#### 3.2.5. Gene Ontology Enrichment Analysis

Similar to the KEGG pathway analysis, GO enrichment analysis was also performed with the targets of the selected miRNAs using the Enrichr tool (Kuleshov et al., 2016), to assess the significance of the roles the selected miRNAs play in different biological activities. The results of different analyses for biological processes, molecular functions, and cellular components are reported in Table 8 and in Supplementary Tables 4, 5, respectively; the details of the enrichment analysis are also given in Supplementary Material. In Table 8, we see that various biological process GO terms which are related to the targets of the 17 selected miRNAs have important roles in cancer development. For example, GO:0023051 is linked with regulation of signaling, which is involved in the development of colorectal cancer. Similarly, GO:0051252 is linked with regulation of the RNA metabolic process of bladder urothelial carcinoma, with an FDR-corrected *p*-value of 1.30E-03, which is <0.05. Other GO terms for biological processes, such as GO:0051171, GO:0048519, and GO:0009653, are linked with the regulation of nitrogen compound metabolic process, negative regulation of biological process, and anatomical structure morphogenesis, respectively, for different cancer types.

**Table 8:** Five significant GO biological processes for each of the 17 selected miRNAs in 10 cancer types.

		**FDR-corrected** **p-value**									
**miRNA**	**GO biological process**	**BLCA**	**BRCA**	**COAD**	**GBM**	**HNSC**	**KIRC**	**LUAD**	**LUSC**	**OV**	**UCEC**
	GO:0051239 regulation of multicellular organismal process	1.30E-04	–	4.34E-05	–	–	–	–	4.70E-04	–	–
	GO:0023051 regulation of signaling	–	–	6.76E-05	–	6.10E-04	–	–	4.70E-04	–	–
hsa-mir-205	GO:0010646 regulation of cell communication	–	–	6.76E-05	–	7.30E-04	–	–	4.70E-04	–	–
	GO:0060255 regulation of macromolecule metabolic process	–	–	–	3.87E-07	–	7.21E-06	–	–	–	7.59E-05
	GO:0051171 regulation of nitrogen compound metabolic process	–	–	–	3.87E-07	–	2.61E-05	–	–	–	7.59E-05
	GO:0006139 nucleobase-containing compound metabolic process	–	8.72E-05	–	–	–	–	2.90E-04	–	1.10E-04	–
	GO:0071840 cellular component organization or biogenesis	2.33E-02	–	–	–	1.39E-06	–	–	–	–	–
hsa-mir-10a	GO:0016043 cellular component organization	2.33E-02	–	–	–	7.28E-05	–	–	–	–	–
	GO:0034641 cellular nitrogen compound metabolic process	–	8.72E-05	–	–	–	–	–	4.60E-03	–	–
	GO:0010467 gene expression	–	1.70E-04	–	–	–	–	–	8.00E-04	–	–
	GO:0048523 negative regulation of cellular process	8.93E-06	–	1.92E-07	2.60E-04	–	6.77E-05	1.30E-04	2.62E-06	1.20E-04	–
	GO:0048519 negative regulation of biological process	2.58E-05	–	1.92E-07	2.60E-04	–	6.77E-05	–	5.64E-06	–	–
hsa-mir-196b	GO:0070482 response to oxygen levels	–	–	–	–	5.06E-06	–	4.43E-05	1.18E-05	1.20E-04	–
	GO:1901700 response to oxygen-containing compound	–	–	–	–	1.00E-04	–	–	1.11E-05	3.28E-05	4.54E-05
	GO:0051173 positive regulation of nitrogen compound metabolic process	2.58E-05	-	1.62E-06	–	–	–	–	–	1.10E-04	–
	GO:0009653 anatomical structure morphogenesis	2.70E-04	–	3.12E-05	–	–	–	–	1.20E-04	–	–
	GO:1901564 organonitrogen compound metabolic process	–	–	–	2.80E-04	9.30E-03	9.50E-03	–	–	–	–
hsa-mir-10b	GO:0048468 cell development	2.50E-04	–	1.10E-04	–	–	–	–	–	–	–
	GO:1903047 mitotic cell cycle process	–	3.30E-03	–	–	2.65E-02	–	–	–	–	–
	GO:0044237 cellular metabolic process	–	–	–	7.10E-04	–	7.00E-03	–	–	–	–
	GO:0048522 positive regulation of cellular process	–	1.25E-05	5.30E-03	–	–	–	5.46E-07	–	5.60E-04	5.50E-03
	GO:0031325 positive regulation of cellular metabolic process	–	1.51E-05	7.60E-03	–	–	–	5.82E-06	–	–	–
hsa-mir-375	GO:1902533 positive regulation of intracellular signal transduction	5.80E-03	–	–	–	–	–	–	–	5.60E-04	–
	GO:0051173 positive regulation of nitrogen compound metabolic process	–	1.51E-05	–	–	3.61E-02	–	–	–	–	–
	GO:0071840 cellular component organization or biogenesis	–	–	8.40E-03	–	–	–	–	–	5.60E-04	–
	GO:0044237 cellular metabolic process	–	2.84E-02	–	–	–	–	–	–	2.30E-03	–
	GO:0008152 metabolic process	–	2.84E-02	–	–	–	–	–	–	8.40E-03	–
hsa-mir-143	GO:1905477 positive regulation of protein localization to membrane	–	2.84E-02	–	–	–	–	–	–	–	–
	GO:1903829 positive regulation of cellular protein localization	–	2.84E-02	–	–	–	–	–	–	–	–
	GO:0006807 nitrogen compound metabolic process	–	2.84E-02	–	–	–	–	–	–	–	–
	GO:0071840 cellular component organization or biogenesis	–	–	4.50E-03	–	6.50E-04	–	–	7.68E-06	–	–
	GO:0016043 cellular component organization	–	–	5.70E-03	–	6.50E-04	–	–	1.49E-05	–	–
hsa-let-7c	GO:0010604 positive regulation of macromolecule metabolic process	–	–	–	3.95E-09	–	–	–	1.20E-04	1.50E-03	–
	GO:0090304 nucleic acid metabolic process	4.30E-04	–	–	–	–	–	–	4.41E-05	–	–
	GO:0051252 regulation of RNA metabolic process	1.30E-03	–	–	–	–	–	–	–	–	2.16E-02
	GO:0048522 positive regulation of cellular process	5.54E-07	5.57E-06	5.00E-03	8.70E-03	–	–	–	1.44E-07	1.40E-04	–
	GO:0048519 negative regulation of biological process	–	–	–	8.70E-03	9.40E-04	3.38E-02	2.88E-06	–	–	–
hsa-mir-107	GO:0051172 negative regulation of nitrogen compound metabolic process	5.54E-07	–	–	–	–	–	2.31E-06	-	6.70E-05	–
	GO:0048518 positive regulation of biological process	1.17E-06	1.07E-05	–	–	–	–	–	2.42E-07	–	–
	GO:0080090 regulation of primary metabolic process	1.31E-06	–	–	–	–	–	–	–	–	9.80E-04
	GO:0044237 cellular metabolic process	–	1.07E-02	–	-	–	–	4.46E-05	2.30E-04	–	–
	GO:1901576 organic substance biosynthetic process	–	1.07E-02	–	–	–	–	4.46E-05	–	–	–
hsa-mir-378	GO:0044249 cellular biosynthetic process	–	1.07E-02	–	–	–	–	4.46E-05	–	–	–
	GO:0034645 cellular macromolecule biosynthetic process	–	1.07E-02	–	–	–	–	–	–	–	1.25E-02
	GO:0009058 biosynthetic process	–	1.07E-02	–	–	–	–	1.45E-05	–	–	–
	GO:0071495 cellular response to endogenous stimulus	–	4.66E-05	–	–	–	2.21E-05	7.70E-04	–	8.10E-03	–
	GO:1901701 cellular response to oxygen-containing compound	–	4.40E-04	–	–	–	3.10E-04	7.70E-04	–	–	–
hsa-mir-133a	GO:0071417 cellular response to organonitrogen compound	–	7.30E-04	–	–	8.70E-03	3.10E-04	–	–	–	–
	GO:0048518 positive regulation of biological process	–	–	–	5.00E-03	8.70E-03	–	–	–	–	9.23E-05
	GO:0065008 regulation of biological quality	–	–	1.50E-03	–	–	–	7.70E-04	–	–	–
	GO:0007049 cell cycle	1.76E-09	8.75E-06	–	–	6.45E-08	–	2.12E-06	4.40E-04	–	9.80E-04
	GO:0051276 chromosome organization	1.52E-07	3.59E-05	–	–	9.12E-07	–	5.41E-07	7.92E-05	–	–
hsa-mir-1	GO:0006261 DNA-dependent DNA replication	4.12E-08	1.40E-04	–	–	–	–	6.25E-07	–	–	–
	GO:0022402 cell cycle process	4.03E-07	–	–	–	2.18E-06	–	–	1.70E-03	–	–
	GO:0006259 DNA metabolic process	–	1.70E-04	–	–	5.77E-06	–	5.41E-07	–	–	–
	GO:0010033 response to organic substance	–	1.03E-02	5.58E-05	–	–	–	–	–	1.97E-05	–
	GO:0060255 regulation of macromolecule metabolic process	3.20E-04	–	–	1.20E-04	–	–	–	–	–	–
hsa-mir-30c	GO:0070887 cellular response to chemical stimulus	–	1.03E-02	2.00E-03	–	–	–	–	–	–	–
	GO:0016192 vesicle-mediated transport	–	1.03E-02	–	–	–	3.60E-03	–	–	–	–
	GO:0002376 immune system process	–	1.03E-02	–	–	–	–	2.05E-05	–	–	–
	GO:0051239 regulation of multicellular organismal process	4.64E-05	5.02E-08	–	–	–	–	–	–	–	–
	GO:0048731 system development	4.10E-04	–	–	–	–	–	5.33E-08	–	–	–
hsa-mir-16	GO:0045595 regulation of cell differentiation	4.10E-04	–	–	–	–	–	2.61E-08	–	–	–
	GO:0050793 regulation of developmental process	–	1.11E-07	–	–	–	–	2.61E-08	–	–	–
	GO:2000026 regulation of multicellular organismal development	–	1.29E-07	–	–	–	–	–	–	1.50E-03	–
	GO:0071840 cellular component organization or biogenesis	–	–	–	–	1.30E-04	–	6.20E-03	1.02E-06	–	2.04E-02
	GO:1903047 mitotic cell cycle process	–	1.99E-02	–	–	–	–	6.20E-03	8.40E-06	–	–
hsa-mir-30a	GO:0090304 nucleic acid metabolic process	–	–	–	6.20E-04	–	–	–	–	–	2.73E-02
	GO:0071310 cellular response to organic substance	–	–	–	–	–	1.90E-03	–	–	3.20E-03	–
	GO:0006260 DNA replication	–	–	–	–	–	–	–	6.33E-06	–	2.04E-02
	GO:0080090 regulation of primary metabolic process	–	–	–	–	–	–	1.35E-02	2.20E-03	–	–
	GO:2000727 positive regulation of cardiac muscle cell differentiation	1.05E-02	–	–	–	–	–	–	–	–	–
hsa-let-7i	GO:1904705 regulation of vascular smooth muscle cell proliferation	1.05E-02	–	–	–	–	–	–	–	–	–
	GO:0071900 regulation of protein serine/threonine kinase activity	1.05E-02	–	–	–	–	–	–	–	–	–
	GO:0061061 muscle structure development	1.05E-02	–	–	–	–	–	–	–	–	–
	GO:0007049 cell cycle	1.13E-13	1.08E-08	–	–	2.65E-11	–	1.14E-08	1.16E-13	–	2.32E-09
	GO:0000278 mitotic cell cycle	1.72E-13	1.08E-08	–	–	4.26E-13	7.08E-05	2.78E-10	1.17E-12	–	–
hsa-mir-24	GO:1903047 mitotic cell cycle process	1.16E-11	4.56E-08	–	–	1.04E-10	7.08E-05	6.94E-10	1.17E-12	–	–
	GO:0022402 cell cycle process	3.15E-11	–	–	–	5.20E-11	9.55E-05	2.75E-07	9.89E-13	–	–
	GO:0044772 mitotic cell cycle phase transition	2.15E-10	4.56E-08	–	–		–	4.86E-10	1.51E-11	–	–
	GO:0060255 regulation of macromolecule metabolic process	3.22E-06	2.03E-05	8.31E-07	6.89E-05	1.30E-03	5.67E-06	2.05E-05	1.88E-06	1.03E-05	9.40E-04
	GO:0080090 regulation of primary metabolic process	3.22E-06	2.03E-05	8.31E-07	–	–	–	5.80E-05	2.99E-06	1.19E-05	9.40E-04
hsa-mir-95	GO:0051171 regulation of nitrogen compound metabolic process	3.22E-06	2.03E-05	8.31E-07	–	–	9.97E-06	5.80E-05	2.99E-06	1.19E-05	–
	GO:0050789 regulation of biological process	9.66E-07	–	1.93E-07	6.89E-05	1.30E-03	9.97E-06	–	–	4.16E-06	–
	GO:0065007 biological regulation	3.22E-06	–	8.31E-07	6.89E-05	1.30E-03	–	–	–	1.19E-05	9.40E-04

As with the biological processes, GO terms for molecular functions were also found to be significant in the development of various types of cancer, as reported in Supplementary Table 4. For example, GO:0008134 is linked with transcription factor binding, which plays an important role in BRCA, GBM, KIRC, LUAD, OV, and UCEC, with respective FDR-corrected *p*-values 8.10E-03, 8.31E-06, 6.80E-06, 4.70E-03, 3.76E-05, and 3.40E-03 all being <0.05. Other important GO terms such as GO:0044877, GO:0003723, GO:0003676, and GO:0008092 are found to be linked to protein-containing complex binding, RNA binding, nucleic acid binding, and cytoskeletal protein binding, respectively, for different cancer types. Supplementary Table 5 reports the significant GO terms for cellular components in various cancer types. GO:0070013 is linked to intracellular organelle lumen, which has FDR-corrected *p*-values within the 5% significance level for seven different cancer types, namely BLCA, BRCA, COAD, GBM, HNSC, KIRC, and LUSC. In addition, GO:0043227, GO:0005654, and GO:0044444 are associated with membrane-bounded organelle, nucleoplasm, and cytoplasmic part, respectively, which are critical processes in the progression of different types of cancer. The relationship between these GO terms and important activities in cancer development have also been cross-validated in other studies (Waldman et al., 1997; Dhillon et al., 2007; He et al., 2013; Reimand et al., 2013; McClurg and Robson, 2015). Taken together, all of these evidences point to the potential importance of the 17 selected miRNAs in the development of various types of cancer.

#### 3.2.6. Protein-Protein Interaction Network Analysis

**Figure 5:**
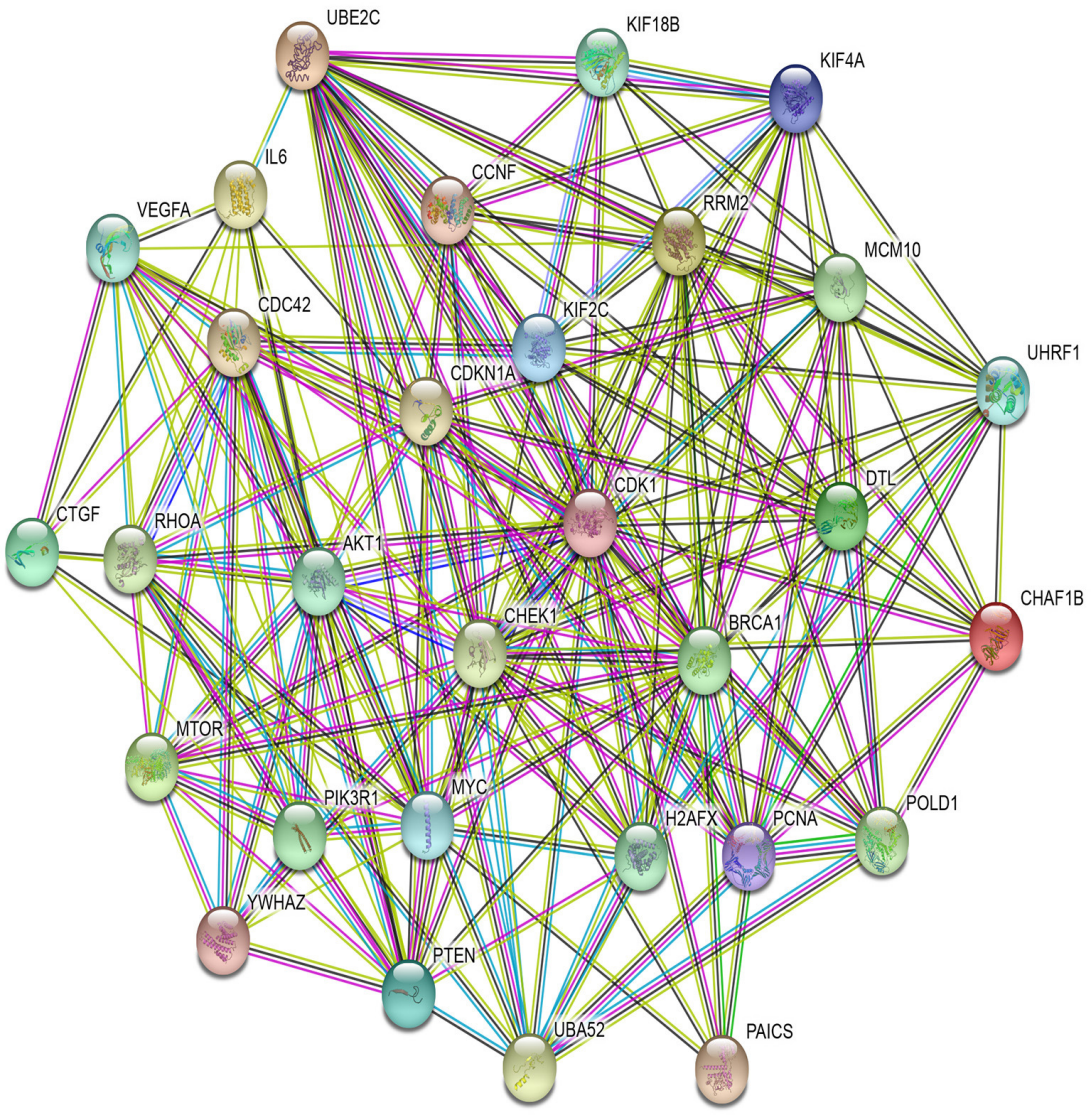
PPI network of top 30 proteins associated with 10 cancer types, with *p*-value <1.0E-16 and average node degree 13.7.

In PPI networks, a node and an edge signify the interaction of a given protein and the protein-protein association. Here, related proteins share common functions, although they do not necessarily physically interact with each other. In our study, to perform the PPI network analysis, 170 sets of targets relating to 17 miRNAs in 10 different cancer types were used to compute the PPI networks using the STRING database (Szklarczyk et al., 2019). Then, the 170 interaction networks were further analyzed to rank the proteins based on the degree of their nodes and their presence in 10 cancer types. The top 30 proteins are reported in Table 9, while the rest are given in Supplementary Material. For example, MYC has degrees 34, 28, 41, 85, 33, 133, 54, 25, 38, and 32 with respect to BLCA, BRCA, COAD, GBM, HNSC, KIRC, LUAD, LUSC, OV, and UCEC, respectively. Therefore, the total degree of MYC is 503. Similarly, other proteins have certain degrees in different cancer types. Moreover, their presence in the different cancer types is indicated by the association count; for example, MYC has an association count of 10. The proteins in Table 9 are used to construct the final consolidated PPI network shown in Figure 5, which represents the associations between the top 30 proteins and 10 different cancer types. The average node degree in this network is 13.7, with PPI enrichment *p* < 1.0E-16. In Figure 5, the significant proteins are MYC, PTEN, CDK1, BRCA1, AKT1, PIK3R1, etc. MYC is the most important protein, known to be oncogenic for breast cancer. Similarly, AKT1 is very significant for breast, lung, and colon cancers. PIK3R1 also plays an important role in breast cancer. The detailed list of PPI networks is provided in Supplementary Material. In summary, the PPI network analysis suggests that the 17 selected miRNAs can be considered important biomarkers with respect to diagnosis of 10 different cancer types.

**Table 9:** Association of top 30 proteins in 10 cancer types for the 17 selected miRNAs through their targets.

	**Node degree of protein in 10 cancer types**										**Total degree**	**Association count**
**TF**	**BLCA**	**BRCA**	**COAD**	**GBM**	**HNSC**	**KIRC**	**LUAD**	**LUSC**	**OV**	**UCEC**		
MYC	34	28	41	85	33	133	54	25	38	32	503	10
VEGFA	23	10	15	53	23	18	36	36	48	39	301	10
AKT1	17	59	50	16	54	32	0	16	17	8	269	9
RRM2	17	28	0	11	21	23	30	32	10	10	182	9
CDK1	23	31	10	13	21	0	21	30	0	24	173	8
CDKN1A	20	19	17	15	15	18	20	17	8	10	159	10
UHRF1	18	29	1	7	21	23	25	14	0	5	143	9
CHEK1	24	22	0	0	21	0	24	28	0	22	141	6
H2AFX	32	16	9	0	10	0	16	21	8	24	136	8
MCM10	20	22	0	0	19	11	20	23	0	18	133	7
POLD1	33	26	0	0	15	14	16	30	0	0	134	6
IL6	11	12	7	29	8	0	15	15	14	16	127	9
RHOA	11	15	9	0	0	8	30	18	22	12	125	8
PCNA	20	26	0	0	0	0	25	40	0	17	128	5
DTL	13	18	0	3	12	13	13	23	10	16	121	9
CCNF	6	18	5	14	7	18	18	14	19	0	119	9
BRCA1	27	12	0	0	25	12	7	25	12	0	120	7
CDC42	8	0	22	17	0	13	12	23	0	19	114	7
PTEN	15	10	7	9	3	0	25	25	18	0	112	8
YWHAZ	5	12	13	9	27	4	11	13	7	3	104	10
PAICS	7	19	0	5	9	0	23	27	1	4	95	8
PIK3R1	7	19	7	7	10	0	16	8	9	5	88	9
UBA52	11	0	8	10	0	16	18	16	0	8	87	7
CTGF	11	12	10	8	5	4	16	13	5	0	84	9
KIF4A	17	15	0	0	10	8	15	11	10	0	86	7
MTOR	9	8	9	0	9	14	7	9	8	8	81	9
UBE2C	13	13	0	0	11	8	12	15	0	11	83	7
KIF2C	16	15	0	0	11	0	16	12	9	0	79	6
KIF18B	11	11	0	0	11	8	11	12	0	11	75	7
CHAF1B	10	13	0	4	12	0	12	9	5	2	67	8

### 3.3. Web Application for Prediction of 10 Cancer Types

A web version of the predictor^4^ was developed for use of the scientific and medical communities to aid in cancer diagnosis. The application uses the random forest and the expression of 17 miRNAs to predict the occurrence of 10 different types of cancer. The random forest was chosen as it outperformed other classifiers in our experiments. To predict the type of cancer for a particular patient or set of patients, the expression values of 17 miRNAs in those patients need to be uploaded in a specific format, instructions are provided on the website. The application will return and display the predicted cancer types for the given set of patients.

## 4. Conclusions

We have proposed a statistical learning method for feature selection that integrates clustering, classification, and regression to find putative miRNAs by solving a multi-class classification problem on 10 cancer types. The first part of the method is a wrapper-based feature selection algorithm, called stochastic covariance evolutionary strategy with forward selection (SCES-FS), which involves stochastic neighbor embedding (SNE), covariance matrix adaptation evolutionary strategy (CMA-ES), forward selection (FS), and a classification technique. Features are reordered using SNE by performing clustering of highly correlated features. From the result of the clustering, a subset of features is randomly selected to perform multi-class classification on 10 cancer types. Although the features are randomly selected, the underlying classification task is treated as an optimization problem for CMA-ES in order to find the features automatically. Thereafter, a final set of features/miRNAs is obtained using forward selection. The results of the first part of SCES-FS have been compared with the results of some well-known feature selection methods, including ESVM-RFE, LASSO, NSGA-II-SE, MOGA, SVM-nRFE, SVM-RFE, CMIM, ICAP, SCAD, JMI, CIFE, mRMR, FSCOX, DISR, SNRs, and RankSum, as well as the result with all features, in terms of classification accuracy. The SCES-FS method selected 17 putative miRNAs associated with 10 cancer types and achieved higher classification accuracy than the other methods. Using the 17 selected miRNAs, a web-based multi-class cancer predictor application has been developed.

These selected miRNAs are used in the second part of the proposed method, which employs Cox regression analysis to examine their importance with respect to survival of particular types of cancer. The analysis uses data on expression of the 17 selected miRNAs together with clinical data. A high Cox coefficient value signifies the importance of an miRNA for a particular cancer type. For example, it is found that hsa-mir-375 has the highest Cox coefficient, 0.9882, for the glioblastoma multiform cancer type. Similar results were obtained for other miRNAs, associated with different cancer types. The up- and down-regulations of the 17 selected miRNAs have been computed based on the ANOVA test. Furthermore, network analysis, expression analysis using hierarchical clustering, KEGG pathway analysis, GO enrichment analysis, and PPI network analysis have been performed to assess the biological significance of the selected miRNAs. The network analysis revealed the association of different cancer types with each pair of miRNA and its target mRNA. The hierarchical clustering analysis demonstrated the effective changes in expression levels of the miRNAs between tumor and normal samples. Both the KEGG and GO enrichment analyses reveals the significant pathways and biological functions in different cancer types. Moreover, using PPI networks, key cancer regulators such as MYC, VEGFA, AKT1, CDKN1A, RHOA, and PTEN are identified. All these evidences suggest that our selected miRNAs play key roles in the development of 10 different types of cancer. A future research direction is the integration of multi-omics data for finding effective regulators pan-cancer biomarkers.

## Data Availability Statement

Publicly available datasets were analyzed in this study. This data can be found here: https://portal.gdc.cancer.gov/ and http://www.nitttrkol.ac.in/indrajit/projects/mirna-prediction-multicalss/.

## Author Contributions

JPS, IS, and AL conceived and designed the experiments. JPS, IS, AL, NG, AD, and PL performed the experiments. JPS, IS, MW, and AD wrote the manuscript. JPS, IS, GB, and DP corrected and edited the manuscript. All authors read and approved the final manuscript.

## Conflict of Interest

JPS was employed by company Larsen & Toubro Infotech Ltd. The remaining authors declare that the research was conducted in the absence of any commercial or financial relationships that could be construed as a potential conflict of interest.

## Footnotes

^1^https://tcga-data.nci.nih.gov/tcga/

^2^http://www.nitttrkol.ac.in/indrajit/projects/mirna-prediction-multicalss/

^3^http://www.nitttrkol.ac.in/indrajit/projects/mirna-prediction-multicalss/

^4^http://www.nitttrkol.ac.in/indrajit/projects/mirna-prediction-multicalss/
