# Supplementary material for: Identification of miRNA Biomarkers for Diverse Cancer Types Using Statistical Learning Methods at the Whole-Genome Scale
Source: Front Genet. 2020 Nov 13;11:982. doi: 10.3389/fgene.2020.00982 (PMC7691578; doi:10.3389/fgene.2020.00982)
Supplement: Supplementary file 1 [file Data_Sheet_1.pdf]

Supplementary Material for “Identification of miRNA Biomarkers for Diverse Cancer Types using Statistical Learning Methods at the Whole Genome Scale”

Jnanendra Prasad Sarkar [1,2,a], **Indrajit Saha** [3,a,*], **Adrian Lancucki** [4,a], **Nimisha Ghosh** [5], **Michal Wlasnowolski** [6], **Grzegorz Bokota** [7,8], **Ashmita Dey** [2], **Piotr Lipinski** [4], **and Dariusz Plewczynski** [6,8,*]

[a] *Equally contributing authors*
[1] *Data, Analytics & AI, Larsen & Toubro Infotech Ltd., Pune, India*
[2] *Department of Computer Science & Engineering, Jadavpur University, Kolkata, India*
[3] *Department of Computer Science and Engineering, National Institute of Technical Teachers' Training and Research, Kolkata, India*
[4] *Computational Intelligence Research Group, Institute of Computer Science, University of Wroclaw, Wroclaw, Poland*
[5] *Department of Computer Science and Information Technology, SOA University, Bhubaneshwar, India*
[6] *Faculty of Mathematics and Information Science, Warsaw University of Technology, Warsaw, Poland*
[7] *Institute of Informatics, University of Warsaw, Warsaw, Poland*
[8] *Centre of New Technologies, University of Warsaw, Warsaw, Poland*

Correspondence*:
Indrajit Saha and Dariusz Plewczynski
indrajit@nitttrkol.ac.in and d.plewczynski@cent.uw.edu.pl

ABSTRACT

Genome-wide analysis of miRNA molecules can reveal important information for understanding the biology of cancer. Typically, miRNAs are used as features in statistical learning methods in order to train learning models to predict cancer. This motivates us to propose a method that integrates clustering and classification techniques for diverse cancer types with survival analysis via regression to identify miRNAs that can potentially play a crucial role in the prediction of different types of tumors. Our method has two parts. The first part is a feature selection procedure, called the stochastic covariance evolutionary strategy with forward selection (SCES-FS), which is developed by integrating stochastic neighbor embedding (SNE), the covariance matrix adaptation evolutionary strategy (CMA-ES), and classifiers, with the primary objective of selecting biomarkers. SNE is used to reorder the features by performing an implicit clustering with highly correlated neighboring features. A subset of features is selected heuristically to perform multi-class classification for diverse cancer types. In the second part of our method, the most important features identified in the first part are used to perform survival analysis via Cox

regression, primarily to examine the effectiveness of the selected features. For this purpose, we have analyzed next generation sequencing data from The Cancer Genome Atlas in form of miRNA expression of 1,707 samples of 10 different cancer types and 333 normal samples. The SCES-FS method is compared with well-known feature selection methods and it is found to perform better in multi-class classification for the 17 selected miRNAs, achieving an accuracy of 96%. Moreover, the biological significance of the selected miRNAs is demonstrated with the help of network analysis, expression analysis using hierarchical clustering, KEGG pathway analysis, GO enrichment analysis, and protein-protein interaction analysis. Overall, the results indicate that the 17 selected QA16 miRNAs are associated with many key cancer regulators, such as MYC, VEGFA, AKT1, CDKN1A, RHOA, and PTEN, through their targets. Therefore the selected miRNAs can be regarded as putative biomarkers for 10 types of cancer.

Keywords: Cancer, Cox Regression, Feature Selection, Gene Ontology, KEGG pathway, Machine Learning, Next Generation Sequencing, Stochastic Neighbor Embedding

1 CLASSIFICATION TECHNIQUES

In this subsection, different classification techniques such as K-Nearest Neighbors (K-NN) Altman (1992), Naive Bayes Classifier (NB) George and Langley (1995), Support Vector Machine (SVM) Cortes and Vapnik (1995), Decision Tree (DT) Quinlan (1986) and Random Forest (RF) Breiman (2005) are discussed.

Among various classifiers, K-Nearest Neighbors (K-NN) Altman (1992) classifier is a simple non-parametric classification method, where a new point is labelled with a label of the majority of its K neighbors. The parameter K is advised to be odd, since it helps in breaking ties.

Naive Bayes (NB) George and Langley (1995) is statistical classifier which uses Bayes' theorem to model a relationship between the attributes and class label. Let $\mathbf{x_i} = (x_{i1}, x_{i2}, \ldots, x_{iD})$ denote features of an observation vector $\mathbf{x} \in \mathbb{R}^D$. Bayes' theorem states the conditional probability between class label y and features x_i as $P(y|x_{i1}, \ldots, x_{iD}) = \frac{P(y)P(x_{i1},\ldots,x_{iD}|y)}{P(x_{i1},\ldots,x_{iD})}$. NB makes a strong assumption on independence of the attributes, which allows to factorize a term in enumerator as $P(y|x_{i1}, \ldots, x_{iD}) = \frac{P(y)\prod_i P(x_i|y)}{P(x_{i1},\ldots,x_{iD})}$. Predicted class label is the one that maximizes $P(y|x_{i1}, \ldots, x_{iD})$ given the input data.

Support Vector Machine (SVM) Cortes and Vapnik (1995) is another popular classifier which performs basically for two-class classification by finding a separating hyperplane $\{\mathbf{x} \in \mathbb{R}^D : \mathbf{w}^T\mathbf{x} + b = 0\}$, which has the highest distance from datapoints of both classes, where $\mathbf{w} \in \mathbb{R}^D$, and $b \in \mathbb{R}$. Extensions to multi-class classification include training one-against-all classifiers for each class separately, or one-against-one for each pair of classes Hsu and Lin (2002). SVM can be extended to non-linear hyperplanes through application of the kernel trick, transforming the data to another space with a kernel function.

Decision Tree (DT) is widely used classifier, where a node corresponds to an input attribute and a set of discrete values, or a threshold splitting the range of possible values in case of continuous variables. Classification trees assign labels to data samples. A new sample is being passed from a root down to a leaf, and leafs hold class labels. Commonly, classification trees are constructed greedily top to bottom, where in every node a variable is chosen along with a split criterion. In the ID3 algorithm Quinlan (1986), the variable with the lowest entropy is greedily chosen as the next node variable. The C4.5 algorithm Quinlan (1993) extends it by, e.g., handling discrete attributes, missing values, or pruning the tree after creation.

On the other hand, Random Forest (RF) Breiman (2005) classifier, consists of a collection of decision trees. Their predictions are combined through majority voting $\mathcal{V}(\mathbf{x}) = \frac{1}{\mathcal{M}} \sum_{i=1}^{\mathcal{M}} \mathcal{T}_i(\mathbf{x})$, where each $\mathcal{T}_i$ denotes the prediction of a separate decision tree classifier. Those individual trees are learned through

bootstrap aggregating, that is selecting with replacement a random sample from the data, and then fitting a classifier to the selected sample.

2 FEATURE SELECTION TECHNIQUES

The applied filter feature selection methods are based on MI, which is a measure of mutual dependence of two random variables given as

$$I(X;Y) = \sum_{y \in Y} \sum_{x \in X} p(x,y) log \left(\frac{p(x,y)}{p(x)p(y)} \right),$$

where $p(x, y)$ is the joint pdf of X and Y, and $p(x), p(y)$ are the marginals. The higher the MI value, the larger the dependence. Other useful relations concerning MI are

$$I(X;Y) = H(X) - H(X|Y),$$

$$I(X;Y) = KL(p(x,y)||p(x)p(y)),$$

where $H()$ denotes entropy, and $KL()$ Kullback-Leibler divergence. Brown et al. Brown et al. (2012a) provide a comprehensive review of those and other methods along with implementations gathered together in the FEAST framework. The Conditional Mutual Information Maximisation (CMIM) Fleuret (2004) method comes as a compromise between the power of the feature alone, and independence of the feature given already selected ones.

The exact formula for CMIM is

$$v(1) = \arg\max_{n} \min_{X_j \in S} I(Y; X_n), \tag{1}$$

$$v(k) = \arg\max_{n} \min_{X_j \in S} I(Y; X_n|X_j), \tag{2}$$

where v returns the index of the next selected feature. The Interaction Capping (ICAP) Jakulin (2005) criterion adds a $\min$ penalty term for feature redundancy

$$J_{ICAP}(X_i) = I(X_i; C) + \sum_{s_j \in S} \min(0, I(X_i; s_j; C)). \tag{3}$$

Joint Mutual Information (JMI) Bennasar et al. (2015) is a generalization of MI for a number of variables:

$$I(X_i, \ldots, X_k; Y) = KL(p(x_i, \ldots, x_k, y)||p(x_i, \ldots, x_k)p(y)).$$

In the original work, it has been used to select inputs for a neural network, and applied with joint pdfs for pairs of variables. In FEAST, it is also applied to pairs, where the final score is computed as

$$J_{jmi} = \sum_{X_j \in S} I(X_k, X_j; Y).$$

Feature selection with Minimum redundancy maximum relevance (mRMR) Peng et al. (2005) is based on mutual information. It maximizes relevance given as

$$D(S,c) = 1/|S| \sum_{x_i \in S} I(x_i;c),$$

where $I(x;c)$ denotes mutual information, S feature set and c a particular class, while minimizing redundancy

$$R(S) = 1/|S|^2 \sum_{x_i,x_j \in S} I(x_i;x_j).$$

As the method works best with categorical data, expression levels have been quantized into 10 equally-spaced bins. Score of the ith feature is computed as

$$SNR_i = \frac{|\mu_i^{(1)} - \mu_i^{(2)}|}{\sigma_i^{(1)} + \sigma_i^{(2)}},$$

where $\mu_i^{(j)}$ denotes mean and $\sigma_i^{(j)}$ variance of class j. Higher values of SNR signify stronger bond with one of the classes. The attributes are sorted in the descending order of their Signal-to-Noise Ratio (SNRs). It is applied greedily over all features, where top k are returned. The test can be applied as a simple method of feature selection, when it works on the data expressed through ranks Troyanskaya et al. (2002). As a feature selection method, it tests equality of data restricted to each of the features alone. For each of the classes, it tests the mean against all other background classes. The other feature selection methods such as Conditional Infomax Feature Extraction (CIFE)and Double Input Symmetrical Relevance (DISR) can be found in Brown et al. (2012b).

3 RESULTS

Table S1. List of 39 miRNAs with their classification accuracy using Random Forest classification

Serial Number	miRNA	Accuracy	Serial Number	miRNA	Accuracy	Serial Number	miRNA	Accuracy	Serial Number	miRNA	Accuracy
1	hsa-mir-205	74.21 ± 0.0384	11	hsa-mir-1	76.88 ± 0.0404	21	hsa-mir-125a	95.34 ± 0.0375	31	hsa-mir-577	82.99 ± 0.0387
2	hsa-mir-10a	74.56 ± 0.0386	12	hsa-mir-30c	78.00 ± 0.0386	22	hsa-mir-152	95.11 ± 0.0372	32	hsa-let-7f	82.55 ± 0.0372
3	hsa-mir-196b	74.78 ± 0.0381	13	hsa-mir-16	79.50 ± 0.0368	23	hsa-mir-101	95.00 ± 0.0379	33	hsa-mir-99a	81.66 ± 0.0389
4	hsa-mir-10b	74.99 ± 0.0374	14	hsa-mir-30a	82.76 ± 0.0403	24	hsa-mir-148a	94.00 ± 0.0405	34	hsa-mir-145	81.22 ± 0.0401
5	hsa-mir-375	75.32 ± 0.0397	15	hsa-let-7i	89.34 ± 0.0397	25	hsa-mir-184	93.77 ± 0.0384	35	hsa-mir-149	80.67 ± 0.0380
6	hsa-mir-143	75.55 ± 0.0397	16	hsa-mir-24	90.56 ± 0.0369	26	hsa-mir-194	93.22 ± 0.0408	36	hsa-mir-326	80.32 ± 0.0394
7	hsa-let-7c	75.73 ± 0.0385	17	hsa-mir-95	96.88 ± 0.0385	27	hsa-mir-628	93.00 ± 0.0402	37	hsa-mir-362	75.89 ± 0.0399
8	hsa-mir-107	75.99 ± 0.0391	18	hsa-mir-27b	96.45 ± 0.0372	28	hsa-mir-28	85.99 ± 0.0366	38	hsa-mir-455	75.55 ± 0.0404
9	hsa-mir-378	76.44 ± 0.0384	19	hsa-mir-135b	96.11 ± 0.0407	29	hsa-mir-139	85.44 ± 0.0386	39	hsa-mir-193a	75.00 ± 0.0370
10	hsa-mir-133a	76.65 ± 0.0389	20	hsa-mir-584	96.00 ± 0.0374	30	hsa-mir-125b	85.00 ± 0.0401			

Table S2. Regulation and FDR of each selected miRNA for ten cancer types

miRNA	BLCA		BRCA		COAD		GBM		HNSC		KIRC		LUAD		LUSC		OV		UCEC	
	Regulation (up↑/down↓)	FDR	Regulation (up↑/down↓)	FDR	Regulation (up↑/down↓)	FDR	Regulation (up↑/down↓)	FDR	Regulation (up↑/down↓)	FDR	Regulation (up↑/down↓)	FDR	Regulation (up↑/down↓)	FDR	Regulation (up↑/down↓)	FDR	Regulation (up↑/down↓)	FDR	Regulation (up↑/down↓)	FDR
hsa-mir-205	↑	1.74E-15	↑	1.44E-06	↓	3.29E-27	↓	5.46E-03	↑	2.23E-56	↓	2.88E-48	-	4.82E-01	↑	1.16E-24	↑	2.08E-03	-	1.05E-01
hsa-mir-10a	↓	1.01E-11	↓	2.46E-09	↑	2.77E-19	↓	1.87E-23	↓	6.42E-72	↓	1.44E-05	↓	1.57E-04	-	9.34E-01	↓	6.35E-121	↑	1.68E-20
hsa-mir-196b	↑	1.29E-09	↑	4.42E-10	↑	2.92E-57	-	8.62E-01	↑	2.50E-02	↑	1.82E-34	-	3.03E-01	↑	5.75E-12	↓	1.42E-03	↑	1.21E-47
hsa-mir-10b	↓	5.28E-07	↓	4.35E-04	↓	2.05E-05	↓	5.39E-21	↓	1.48E-18	↑	1.72E-02	↓	6.00E-17	↓	4.08E-12	↓	2.27E-116	↓	4.71E-03
hsa-mir-375	↓	3.90E-14	↑	6.40E-50	↑	4.59E-46	↓	2.36E-14	↓	3.96E-14	↓	4.87E-42	↑	2.02E-21	-	4.24E-01	↓	2.00E-98	↑	1.25E-11
hsa-mir-143	↑	8.32E-05	↓	1.32E-65	↓	1.82E-02	↓	1.87E-23	↓	1.14E-04	↓	3.78E-16	↓	6.28E-09	↓	4.98E-09	↓	1.79E-56	↓	1.07E-27
hsa-let-7c	↓	1.31E-37	↓	3.89E-37	↓	5.88E-58	↑	6.32E-16	↓	1.83E-45	↓	7.84E-45	↓	2.84E-31	↓	2.53E-27	↓	5.14E-29	↓	4.57E-27
hsa-mir-107	↑	7.95E-49	↑	2.71E-95	↓	7.99E-03	↑	1.87E-23	↑	1.18E-103	↑	2.69E-67	↑	1.14E-34	↑	7.17E-47	↑	1.13E-131	↑	1.84E-50
hsa-mir-378	↓	3.19E-17	↓	8.75E-79	↑	2.89E-06	↓	7.79E-20	↓	8.63E-29	↓	2.23E-42	↓	2.01E-31	↓	9.98E-34	↓	1.04E-64	↓	6.39E-39
hsa-mir-133a	↑	1.21E-22	↑	1.35E-22	↑	1.93E-36	↑	4.73E-20	↑	4.05E-54	↑	5.08E-25	↑	1.71E-13	↑	3.75E-14	↑	5.13E-99	↑	5.81E-22
hsa-mir-1	↓	1.38E-03	↓	3.26E-60	-	5.45E-01	↑	1.04E-07	-	8.11E-01	↓	3.74E-27	↓	1.22E-07	↓	1.77E-15	-	1.84E-01	↓	1.34E-11
hsa-mir-30c	↓	3.16E-35	-	1.61E-01	↓	6.34E-17	↓	2.93E-13	↓	1.39E-80	↑	4.82E-08	↓	1.12E-14	↓	8.70E-30	↓	4.02E-04	-	1.72E-01
hsa-mir-16	↑	7.95E-49	↑	2.71E-95	↑	4.31E-58	↑	1.87E-23	↑	1.18E-103	↑	2.69E-67	↑	1.14E-34	↑	7.17E-47	↑	1.79E-132	↑	1.84E-50
hsa-mir-30a	↓	2.62E-44	↓	2.21E-06	↓	9.80E-56	↓	2.14E-23	↓	1.09E-95	↑	6.97E-18	↓	4.41E-17	↓	9.12E-34	↓	1.79E-132	↓	2.52E-17
hsa-let-7i	↓	2.37E-12	↑	2.83E-43	↓	6.17E-25	↑	2.69E-21	↑	6.45E-07	↑	3.02E-38	↑	4.98E-12	↑	8.27E-08	↑	3.25E-125	-	4.71E-01
hsa-mir-24	↓	7.95E-49	↓	2.71E-95	↓	4.31E-58	↓	1.87E-23	↓	1.18E-103	↓	2.69E-67	↓	1.14E-34	↓	7.17E-47	↓	1.79E-132	↓	1.84E-50
hsa-mir-95	↓	8.18E-07	↑	3.91E-11	↑	3.60E-44	↑	1.87E-23	↑	4.87E-22	↑	2.77E-09	↑	3.27E-04	-	3.79E-01	↑	2.75E-125	↑	1.39E-13

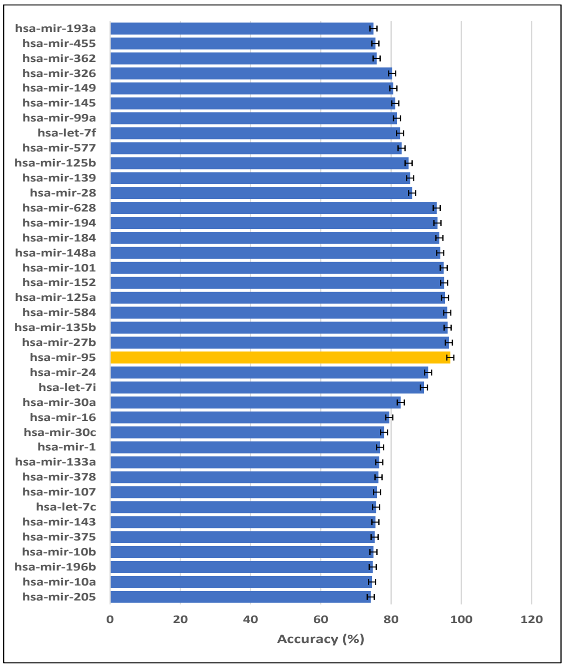

Figure S1. Barchart representation of classification accuracy (RF) of miRNAs for performing multi-class classification of ten diverse cancer types. The higher accuracy is archived by considering 39 miRNAs and that has been marked with yellow color

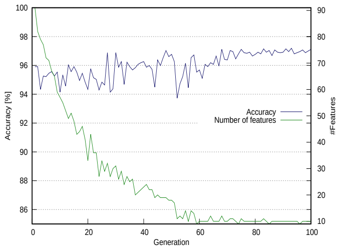

(a)

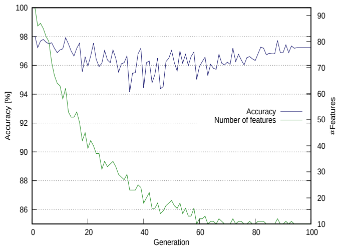

(b)

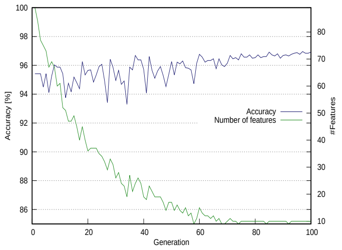

(c)

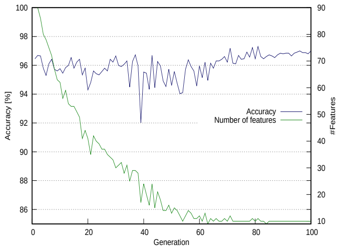

(d)

Figure S2. Performance of the proposed SCES feature selection method with (a) RF, (b) SVM, (c) NB and (d) K-NN classifiers and representation based on SNE. The figures depict single SCES runs. Classification accuracy varies during rapid decrease of the number of features, and converges near the end after nearly 90 generations

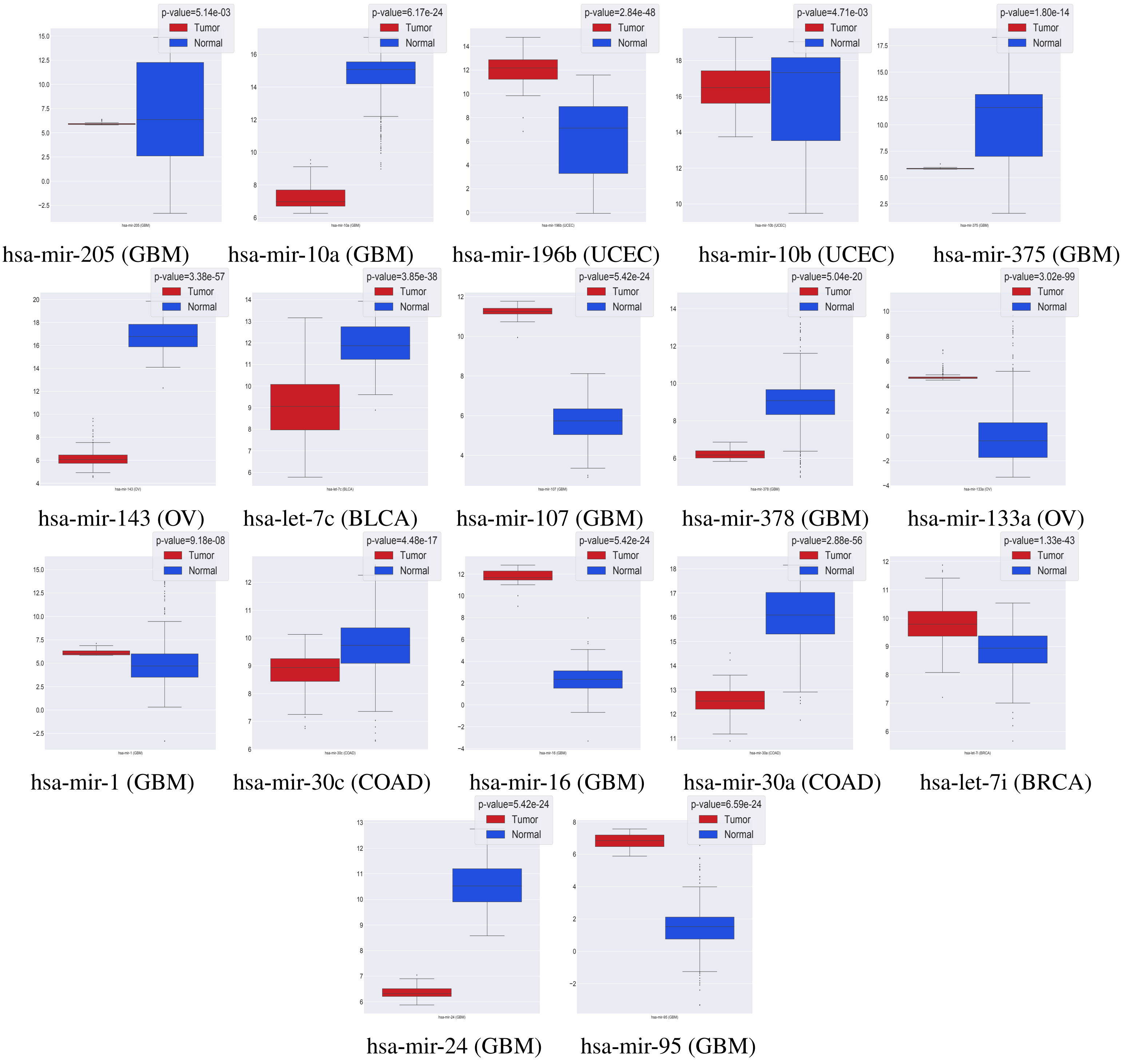

Figure S3. Expression analysis using box plots of expression values for hsa-mir-205 (GBM), hsa-mir-10a (GBM), hsa-mir-196b (UCEC), hsa-mir-10b (UCEC), hsa-mir-375 (GBM), hsa-mir-143 (OV), hsa-let-7c (BLCA), hsa-mir-107 (GBM), hsa-mir-378 (GBM), hsa-mir-133a (OV), hsa-mir-1 (GBM), hsa-mir-30c (COAD), hsa-mir-16 (GBM), hsa-mir-30a (COAD), hsa-let-7i (BRCA), hsa-mir-24 (GBM) and hsa-mir-95 (GBM)

This is a provisional file, not the final typeset article

Table S3 Association of selected 17 miRNAs and their targets in ten diverse cancer types

miRNA-targets	Association in different cancer types										Association Count	Cumulative Corr. Score
	BLCA	BRCA	COAD	GBM	HNSC	KIRC	LUAD	LUSC	OV	UCEC		
hsa-mir-205-CYR61	✓	-	✓	-	-	-	-	✓	-	-	3	-1.716
hsa-mir-205-CTGF	✓	-	✓	-	-	-	-	✓	-	-	3	-1.653
hsa-mir-205-TNFSF8	✓	-	✓	-	-	-	-	✓	-	-	3	-1.621
hsa-mir-205-SLC7A2	-	-	✓	-	-	-	-	-	-	✓	2	-1.517
hsa-mir-205-PDLIM5	-	-	✓	✓	-	-	-	-	-	-	2	-1.379
hsa-mir-205-STARD8	✓	-	-	-	-	-	-	✓	-	-	2	-1.311
hsa-mir-205-TIMP1	-	-	✓	-	-	-	-	-	-	-	1	-0.963
hsa-mir-205-SESN3	-	-	✓	-	-	-	-	-	-	-	1	-0.943
hsa-mir-205-SAMD8	-	-	✓	-	-	-	-	-	-	-	1	-0.899
hsa-mir-10a-TTYH3	-	-	✓	-	-	✓	-	-	-	-	2	-1.261
hsa-mir-10a-LILRA2	-	-	✓	-	-	✓	-	-	-	-	2	-1.220
hsa-mir-10a-CARHSP1	-	-	✓	-	-	✓	-	-	-	-	2	-1.197
hsa-mir-10a-CD3D	-	-	✓	-	-	✓	-	-	-	-	2	-1.176
hsa-mir-10a-H3F3C	-	-	✓	-	-	-	-	-	-	-	1	-0.962
hsa-mir-10a-FHL2	-	-	✓	-	-	-	-	-	-	-	1	-0.911
hsa-mir-10a-MTR	-	-	✓	-	-	-	-	-	-	-	1	-0.899
hsa-mir-10a-SFT2D2	-	-	✓	-	-	-	-	-	-	-	1	-0.882
hsa-mir-196b-GATA6	✓	-	✓	-	-	-	-	✓	-	-	3	-1.877
hsa-mir-196b-LAMB2	-	-	✓	-	-	-	-	✓	-	-	2	-1.139
hsa-mir-196b-MAP2K2	-	-	✓	✓	-	-	-	-	-	-	2	-1.124
hsa-mir-196b-KCTD21	-	-	✓	-	-	-	-	-	-	-	1	-0.989
hsa-mir-196b-ACER2	-	-	✓	-	-	-	-	-	-	-	1	-0.974
hsa-mir-196b-BCAR3	-	-	✓	-	-	-	-	-	-	-	1	-0.968
hsa-mir-196b-IGF2BP3	-	-	✓	-	-	-	-	-	-	-	1	-0.943
hsa-mir-196b-HIST1H2BD	-	-	✓	-	-	-	-	-	-	-	1	-0.910
hsa-mir-196b-CDK5R1	-	-	✓	-	-	-	-	-	-	-	1	-0.903
hsa-mir-10b-POC1A	-	✓	✓	-	-	-	-	-	-	-	2	-1.392

Continued on next page

Table S3 – continued from previous page

miRNA-mRNA	Association in 10 diverse cancer types										Association Count	Cumulative Corr. Score
	BLCA	BRCA	COAD	GBM	HNSC	KIRC	LUAD	LUSC	OV	UCEC		
hsa-mir-10b-UHRF1	-	✓	✓	-	-	-	-	-	-	-	2	-1.236
hsa-mir-10b-TUBA1B	-	✓	-	-	-	✓	-	-	-	-	2	-1.106
hsa-mir-10b-INHBA	-	-	✓	-	-	-	-	-	-	-	1	-0.991
hsa-mir-10b-MBNL3	-	-	✓	-	-	-	-	-	-	-	1	-0.982
hsa-mir-10b-OPA3	-	-	✓	-	-	-	-	-	-	-	1	-0.961
hsa-mir-10b-SLC2A3	-	-	✓	-	-	-	-	-	-	-	1	-0.955
hsa-mir-10b-PPP1R13B	-	-	✓	-	-	-	-	-	-	-	1	-0.935
hsa-mir-10b-CD3D	-	-	✓	-	-	-	-	-	-	-	1	-0.928
hsa-mir-375-CRIM1	-	-	✓	-	-	-	✓	-	-	-	2	-1.306
hsa-mir-375-CFL2	-	✓	✓	-	-	-	-	-	-	-	2	-1.258
hsa-mir-375-RHOQ	-	✓	✓	-	-	-	-	-	-	-	2	-1.258
hsa-mir-375-TNS1	-	✓	✓	-	-	-	-	-	-	-	2	-1.204
hsa-mir-375-SON	-	-	✓	-	-	-	-	-	-	-	1	-0.994
hsa-mir-375-LIMD2	-	-	✓	-	-	-	-	-	-	-	1	-0.981
hsa-mir-375-ATG7	-	-	✓	-	-	-	-	-	-	-	1	-0.980
hsa-mir-375-JAK2	-	-	✓	-	-	-	-	-	-	-	1	-0.958
hsa-mir-143-RAB10	-	-	✓	-	-	-	✓	-	-	-	2	-1.264
hsa-mir-143-CENPM	-	✓	✓	-	-	-	-	-	-	-	2	-1.144
hsa-mir-143-NKPD1	-	-	✓	-	-	-	-	-	-	-	1	-0.987
hsa-mir-143-TIAL1	-	-	✓	-	-	-	-	-	-	-	1	-0.981
hsa-mir-143-TUBD1	-	-	✓	-	-	-	-	-	-	-	1	-0.951
hsa-mir-143-PHAX	-	-	✓	-	-	-	-	-	-	-	1	-0.948
hsa-mir-143-TTC38	-	-	✓	-	-	-	-	-	-	-	1	-0.944
hsa-mir-143-FADS6	-	-	✓	-	-	-	-	-	-	-	1	-0.936
hsa-let-7c-LDHA	-	-	✓	-	✓	-	✓	-	-	-	3	-1.951
hsa-let-7c-MRPL12	-	✓	✓	-	-	-	✓	-	-	-	3	-1.883
hsa-let-7c-BRI3BP	-	✓	✓	-	-	-	✓	-	-	-	3	-1.863
hsa-let-7c-YWHAZ	-	-	✓	-	-	-	✓	-	-	-	2	-1.643
hsa-let-7c-E2F5	-	-	✓	-	-	-	✓	-	-	-	2	-1.466

Continued on next page

This is a provisional file, not the final typeset article

Table S3 – continued from previous page

miRNA-mRNA	Association in 10 diverse cancer types										Association Count	Cumulative Corr. Score
	BLCA	BRCA	COAD	GBM	HNSC	KIRC	LUAD	LUSC	OV	UCEC		
hsa-let-7c-PTMA	-	-	✓	-	-	-	✓	-	-	-	2	-1.356
hsa-let-7c-HMGXB4	-	-	✓	-	-	-	-	-	-	-	1	-0.981
hsa-let-7c-HES5	-	-	✓	-	-	-	-	-	-	-	1	-0.980
hsa-let-7c-WDR3	-	-	✓	-	-	-	-	-	-	-	1	-0.967
hsa-mir-107-CPEB3	✓	-	-	-	✓	✓	✓	✓	-	-	5	-3.224
hsa-mir-107-TGFBR3	✓	✓	-	-	-	✓	✓	-	-	-	4	-2.958
hsa-mir-107-FGF2	✓	✓	✓	-	-	-	✓	-	-	-	4	-2.861
hsa-mir-107-CAV1	-	✓	-	-	-	-	✓	✓	-	-	3	-2.285
hsa-mir-107-KLF4	✓	✓	-	-	-	-	✓	-	-	-	3	-2.089
hsa-mir-107-RS1	-	-	-	-	-	-	✓	✓	-	-	2	-1.763
hsa-mir-107-SH3GL2	-	-	-	-	-	✓	✓	-	-	-	2	-1.695
hsa-mir-107-TMEM87A	-	-	✓	-	-	-	-	-	-	-	1	-0.963
hsa-mir-107-CDC42SE2	-	-	✓	-	-	-	-	-	-	-	1	-0.952
hsa-mir-378-SERPINH1	-	-	✓	-	✓	-	✓	-	-	-	3	-1.907
hsa-mir-378-HIST1H2BD	-	✓	✓	-	-	-	✓	-	-	-	3	-1.828
hsa-mir-378-NWD1	-	-	✓	✓	-	-	-	-	-	-	2	-1.552
hsa-mir-378-NME4	-	-	✓	-	-	-	✓	-	-	-	2	-1.505
hsa-mir-378-ORAI2	-	✓	✓	-	-	-	-	-	-	-	2	-1.480
hsa-mir-378-TMEM154	-	-	✓	-	-	-	-	-	-	-	1	-0.976
hsa-mir-378-MYADM	-	-	✓	-	-	-	-	-	-	-	1	-0.969
hsa-mir-378-OPA3	-	-	✓	-	-	-	-	-	-	-	1	-0.957
hsa-mir-133a-TMEM59	-	-	✓	-	-	✓	-	-	-	-	2	-1.523
hsa-mir-133a-KRT7	-	-	✓	-	-	✓	-	-	-	-	2	-1.388
hsa-mir-133a-TCTEX1D2	-	-	✓	-	-	✓	-	-	-	-	2	-1.280
hsa-mir-133a-PNP	-	-	✓	-	-	✓	-	-	-	-	2	-1.192
hsa-mir-133a-UGT2B10	-	-	✓	-	-	-	-	-	-	-	1	-0.930
hsa-mir-133a-CDC42	-	-	✓	-	-	-	-	-	-	-	1	-0.908
hsa-mir-133a-SEC61B	-	-	✓	-	-	-	-	-	-	-	1	-0.903
hsa-mir-133a-MYL12A	-	-	✓	-	-	-	-	-	-	-	1	-0.803

Continued on next page

Table S3 – continued from previous page

miRNA-mRNA	Association in 10 diverse cancer types										Association Count	Cumulative Corr. Score
	BLCA	BRCA	COAD	GBM	HNSC	KIRC	LUAD	LUSC	OV	UCEC		
hsa-mir-1-SRP19	✓	-	✓	-	-	-	-	-	-	-	2	-1.515
hsa-mir-1-GNG5	✓	-	✓	-	-	-	-	-	-	-	2	-1.456
hsa-mir-1-CAPZA1	✓	-	✓	-	-	-	-	-	-	-	2	-1.402
hsa-mir-1-DPY30	✓	-	✓	-	-	-	-	-	-	-	2	-1.373
hsa-mir-1-BMP7	-	-	✓	-	-	-	-	-	-	-	1	-0.988
hsa-mir-1-CAST	-	-	✓	-	-	-	-	-	-	-	1	-0.973
hsa-mir-1-CEBPA	-	-	✓	-	-	-	-	-	-	-	1	-0.972
hsa-mir-1-GOLGA7	-	-	✓	-	-	-	-	-	-	-	1	-0.967
hsa-mir-30c-UBE2I	-	-	✓	-	-	✓	-	-	-	-	2	-1.456
hsa-mir-30c-VIM	-	-	✓	-	-	✓	-	-	-	-	2	-1.349
hsa-mir-30c-SERPINE1	-	-	✓	-	-	✓	-	-	-	-	2	-1.327
hsa-mir-30c-LHFPL2	-	-	✓	-	-	✓	-	-	-	-	2	-1.301
hsa-mir-30c-SOCS3	-	-	✓	-	-	-	-	-	-	-	1	-0.978
hsa-mir-30c-ARF3	-	-	✓	-	-	-	-	-	-	-	1	-0.949
hsa-mir-30c-FOXA1	-	-	✓	-	-	-	-	-	-	-	1	-0.948
hsa-mir-30c-CADPS2	-	-	✓	-	-	-	-	-	-	-	1	-0.843
hsa-mir-16-PHYHIP	✓	✓	✓	✓	-	✓	-	-	-	✓	6	-4.142
hsa-mir-16-CPEB3	✓	✓	-	-	✓	✓	✓	✓	-	-	6	-3.825
hsa-mir-16-DIXDC1	✓	✓	✓	-	-	-	✓	✓	-	-	5	-3.715
hsa-mir-16-RUNX1T1	✓	✓	✓	-	-	-	✓	✓	-	-	5	-3.642
hsa-mir-16-SLC6A4	-	-	-	-	-	✓	✓	✓	-	✓	4	-3.172
hsa-mir-16-CTDSPL	-	-	✓	-	-	✓	✓	✓	-	-	4	-3.003
hsa-mir-16-RS1	-	-	✓	-	-	-	✓	✓	-	-	3	-2.485
hsa-mir-16-OSCAR	-	-	✓	-	-	-	✓	✓	-	-	3	-2.393
hsa-mir-16-STK33	-	-	✓	-	-	✓	-	-	-	-	2	-1.697
hsa-mir-16-FLNA	✓	-	✓	-	-	-	-	-	-	-	2	-1.660
hsa-mir-16-CLIP2	-	-	✓	-	-	-	-	-	-	-	1	-0.996
hsa-mir-16-CAMSAP1	-	-	✓	-	-	-	-	-	-	-	1	-0.995
hsa-mir-30a-CDC20	-	-	✓	-	-	✓	✓	✓	-	-	4	-2.470

Continued on next page

This is a provisional file, not the final typeset article

Table S3 – continued from previous page

miRNA-mRNA	Association in 10 diverse cancer types										Association Count	Cumulative Corr. Score
	BLCA	BRCA	COAD	GBM	HNSC	KIRC	LUAD	LUSC	OV	UCEC		
hsa-mir-30a-MYBL2	-	-	✓	-	-	✓	✓	✓	-	-	4	-2.332
hsa-mir-30a-SFXN1	-	-	✓	-	-	-	✓	✓	-	-	3	-2.089
hsa-mir-30a-MTHFD2	-	-	✓	-	-	✓	✓	-	-	-	3	-1.772
hsa-mir-30a-SOX12	-	-	✓	-	-	-	✓	-	-	-	2	-1.439
hsa-mir-30a-PES1	-	-	✓	✓	-	-	-	-	-	-	2	-1.389
hsa-mir-30a-NCEH1	-	-	✓	-	-	-	-	-	-	-	1	-0.964
hsa-mir-30a-SBF1	-	-	✓	-	-	-	-	-	-	-	1	-0.961
hsa-let-7i-TUBB2A	-	-	✓	-	-	✓	-	-	-	-	2	-1.567
hsa-let-7i-MSI2	-	-	✓	-	-	✓	-	-	-	-	2	-1.491
hsa-let-7i-RAB11FIP4	-	-	✓	-	-	✓	-	-	-	-	2	-1.383
hsa-let-7i-PRSS22	-	-	✓	-	-	✓	-	-	-	-	2	-1.306
hsa-let-7i-SLC20A1	-	-	✓	-	-	-	-	-	-	-	1	-0.929
hsa-let-7i-SURF4	-	-	✓	-	-	-	-	-	-	-	1	-0.886
hsa-let-7i-ACOT9	-	-	✓	-	-	-	-	-	-	-	1	-0.867
hsa-let-7i-ZNF200	-	-	✓	-	-	-	-	-	-	-	1	-0.838
hsa-mir-24-C1QTNF6	✓	✓	-	-	✓	✓	✓	✓	-	-	6	-3.906
hsa-mir-24-PKMYT1	✓	✓	-	-	✓	✓	✓	✓	-	-	6	-3.873
hsa-mir-24-UBE2C	✓	✓	-	-	-	✓	✓	✓	-	-	5	-3.500
hsa-mir-24-RRM2	✓	✓	-	-	-	✓	✓	✓	-	-	5	-3.357
hsa-mir-24-CCNB1	✓	✓	-	-	-	-	✓	✓	-	-	4	-2.825
hsa-mir-24-CDK1	✓	✓	-	-	-	-	✓	✓	-	-	4	-2.780
hsa-mir-24-NEK6	-	-	✓	-	-	✓	✓	-	-	-	3	-1.995
hsa-mir-24-ALDOA	-	✓	-	-	-	✓	✓	-	-	-	3	-1.995
hsa-mir-24-IGSF6	-	-	✓	-	-	✓	-	-	-	-	2	-1.693
hsa-mir-24-CLEC7A	-	-	✓	-	-	✓	-	-	-	-	2	-1.672
hsa-mir-24-TTLL7	-	-	✓	-	-	-	-	-	-	-	1	-0.987
hsa-mir-24-DCAF4	-	-	✓	-	-	-	-	-	-	-	1	-0.979
hsa-mir-95-TBX18	-	-	✓	-	-	-	-	-	-	-	1	-0.974
hsa-mir-95-MRAS	-	-	✓	-	-	-	-	-	-	-	1	-0.852

Continued on next page

Table S3 – continued from previous page

miRNA-mRNA	Association in 10 diverse cancer types										Association Count	Cumulative Corr. Score
	BLCA	BRCA	COAD	GBM	HNSC	KIRC	LUAD	LUSC	OV	UCEC		
hsa-mir-95-ACVR1	-	-	✓	-	-	-	-	-	-	-	1	-0.839
hsa-mir-95-WARS	-	-	✓	-	-	-	-	-	-	-	1	-0.815
hsa-mir-95-ARPC1B	-	-	✓	-	-	-	-	-	-	-	1	-0.810
hsa-mir-95-ARHGDIA	-	-	✓	-	-	-	-	-	-	-	1	-0.804
hsa-mir-95-SYT11	-	-	✓	-	-	-	-	-	-	-	1	-0.787
hsa-mir-95-P2RY1	-	-	✓	-	-	-	-	-	-	-	1	-0.769
hsa-mir-95-TMEM200C	-	-	✓	-	-	-	-	-	-	-	1	-0.707

Table S4. *Five* significant GO Molecular Function for each selected 17 miRNAs in ten diverse cancer types

miRNA	GO-Molecular Function	FDR corrected *p-value*									
		BLCA	BRCA	COAD	GBM	HNSC	KIRC	LUAD	LUSC	OV	UCEC
hsa-mir-205	GO:0008134 transcription factor binding	-	8.10E-03	-	8.31E-06	-	6.80E-06	4.70E-03	-	3.76E-05	3.40E-03
	GO:0044877 protein-containing complex binding	-	-	-	4.40E-03	-	-	4.70E-03	2.20E-03	-	1.19E-02
	GO:0005161 platelet-derived growth factor receptor binding	-	1.64E-02	-	-	-	-	-	1.10E-03	-	1.14E-02
	GO:0019899 enzyme binding	-	-	3.01E-02	-	-	-	4.70E-03	3.30E-03	-	-
	GO:0061629 RNA polymerase II-specific DNA-binding transcription factor binding	-	1.56E-02	-	-	-	-	-	-	7.00E-03	-
hsa-mir-10a	GO:1901363 heterocyclic compound binding	-	4.85E-02	-	-	-	-	2.00E-03	6.30E-03	1.10E-03	-
	GO:0097159 organic cyclic compound binding	-	4.85E-02	-	-	-	-	2.00E-03	6.30E-03	1.10E-03	-
	GO:0003723 RNA binding	-	4.85E-02	-	-	-	-	-	6.30E-03	-	-
	GO:0003676 nucleic acid binding	-	4.85E-02	-	-	-	-	-	-	2.57E-02	-
	GO:0016881 acid-amino acid ligase activity	-	4.85E-02	-	-	-	-	-	-	-	-
hsa-mir-196b	GO:0019899 enzyme binding	8.37E-07	5.80E-04	6.76E-07	-	3.51E-06	4.68E-05	8.22E-07	9.69E-09	8.42E-07	1.33E-05
	GO:0019900 kinase binding	3.00E-06	5.80E-04	-	-	3.51E-06	-	9.86E-05	6.72E-08	6.74E-05	1.00E-04
	GO:0019901 protein kinase binding	1.31E-05	1.70E-03	-	-	1.36E-05	5.56E-05	4.20E-04	2.00E-06	-	-
	GO:1990837 sequence-specific double-stranded DNA binding	-	-	1.48E-06	2.90E-04	-	5.56E-05	4.20E-04	-	-	1.20E-03
	GO:0003690 double-stranded DNA binding	1.10E-04	-	6.76E-07	1.10E-04	-	-	-	-	-	8.70E-04
hsa-mir-10b	GO:0005488 binding	5.20E-03	-	-	2.79E-02	-	-	3.40E-02	-	-	1.11E-02
	GO:0005515 protein binding	5.20E-03	-	-	3.02E-02	-	-	-	-	8.00E-04	-
	GO:1901363 heterocyclic compound binding	-	4.00E-04	-	-	-	-	3.40E-02	-	-	-
	GO:0097159 organic cyclic compound binding	-	4.00E-04	-	-	-	-	3.40E-02	-	-	-
	GO:0001221 transcription cofactor binding	7.80E-03	-	-	-	-	-	-	-	-	-
hsa-mir-375	GO:0005515 protein binding	1.11E-02	1.20E-03	9.58E-05	-	-	-	3.08E-07	-	1.50E-03	3.05E-05
	GO:0005488 binding	-	3.10E-03	-	-	-	-	2.40E-03	-	-	3.00E-03
	GO:0019904 protein domain specific binding	-	-	1.49E-02	-	-	-	1.01E-02	-	5.60E-03	-
	GO:0043548 phosphatidylinositol 3-kinase binding	8.00E-04	4.97E-02	-	-	-	-	-	-	-	-
	GO:0005010 insulin-like growth factor-activated receptor activity	1.11E-02	-	-	-	-	-	-	-	-	1.98E-02
hsa-mir-143	GO:0048407 platelet-derived growth factor binding	-	-	-	-	-	-	1.51E-02	-	-	-
	GO:1901363 heterocyclic compound binding	-	-	-	-	-	-	-	-	6.50E-03	-
	GO:0097159 organic cyclic compound binding	-	-	-	-	-	-	-	-	6.50E-03	-
	GO:0043167 ion binding	-	-	-	-	-	-	-	-	3.49E-02	-
hsa-let-7c	GO:0008134 transcription factor binding	-	-	-	7.58E-06	-	-	-	5.70E-03	-	-
	GO:0000979 RNA polymerase II core promoter sequence-specific DNA binding	-	-	-	-	-	2.03E-02	-	2.59E-02	-	-
	GO:1990841 promoter-specific chromatin binding	-	2.67E-02	-	-	-	-	-	-	-	-
	GO:0003682 chromatin binding	-	2.67E-02	-	-	-	-	-	-	-	-
	GO:1901363 heterocyclic compound binding	-	2.89E-02	-	-	-	-	-	-	-	-
hsa-mir-107	GO:0008134 transcription factor binding	1.03E-02	4.60E-02	-	-	-	-	3.30E-02	-	-	-
	GO:0000900 translation repressor activity, mRNA regulatory element binding	-	4.60E-02	-	-	-	-	3.30E-02	3.11E-02	-	-
	GO:0140096 catalytic activity, acting on a protein	1.03E-02	4.60E-02	-	-	-	-	-	-	-	-
	GO:0016773 phosphotransferase activity, alcohol group as acceptor	1.03E-02	-	-	-	-	-	-	3.11E-02	-	-
	GO:0005515 protein binding	2.00E-03	-	-	-	-	-	-	-	-	-
hsa-mir-378	GO:0003676 nucleic acid binding	1.64E-06	-	-	-	-	-	1.60E-02	-	-	8.00E-03
	GO:1901363 heterocyclic compound binding	2.54E-06	-	-	-	-	-	-	2.30E-02	-	-
	GO:0097159 organic cyclic compound binding	2.89E-06	-	-	-	-	-	-	2.30E-02	-	-
	GO:0003677 DNA binding	9.80E-04	-	-	-	-	-	-	-	-	1.87E-02
	GO:0003723 RNA binding	8.70E-03	-	-	-	-	-	1.60E-02	-	-	-
hsa-mir-133a	GO:0043167 ion binding	-	6.50E-03	-	2.37E-02	-	-	1.82E-02	4.32E-02	-	-
	GO:0005515 protein binding	-	-	-	1.26E-02	-	-	1.82E-02	4.93E-02	-	1.11E-02
	GO:0032427 GBD domain binding	-	-	-	1.79E-02	-	-	1.82E-02	4.32E-02	-	2.12E-02
	GO:0005516 calmodulin binding	-	3.80E-03	-	-	-	-	1.82E-02	4.93E-02	-	-
	GO:0035091 phosphatidylinositol binding	-	4.50E-03	-	-	3.34E-02	-	1.82E-02	-	-	-
hsa-mir-1	GO:1901363 heterocyclic compound binding	5.70E-04	-	-	1.84E-02	-	-	1.10E-04	-	-	1.97E-02
	GO:0097159 organic cyclic compound binding	5.70E-04	-	-	1.84E-02	-	-	1.30E-04	-	-	1.97E-02
	GO:0005524 ATP binding	5.70E-04	-	-	-	-	-	-	1.20E-03	-	-
	GO:0005515 protein binding	-	3.85E-02	-	-	-	-	5.95E-09	-	-	-
	GO:0005488 binding	-	-	-	4.90E-04	-	-	8.49E-05	-	-	-
hsa-mir-30c	GO:0005515 protein binding	1.00E-04	-	1.28E-05	-	2.10E-03	7.60E-04	3.80E-03	-	1.27E-05	8.10E-03
	GO:0005488 binding	2.16E-02	-	1.28E-05	5.93E-05	7.30E-03	-	-	-	1.10E-03	-
	GO:0019899 enzyme binding	2.16E-02	-	-	-	-	-	3.90E-04	-	2.42E-05	-
	GO:0042802 identical protein binding	5.10E-03	-	3.70E-03	-	-	-	-	-	-	-
	GO:0098772 molecular function regulator	-	-	-	-	3.14E-02	-	-	-	3.80E-04	-
hsa-mir-16	GO:0005488 binding	1.70E-04	-	-	-	1.42E-02	-	1.30E-03	-	1.70E-03	1.11E-02
	GO:0046332 SMAD binding	5.70E-04	-	-	-	-	-	4.70E-04	7.70E-03	-	-
	GO:0005515 protein binding	7.40E-04	3.70E-03	-	-	-	-	2.90E-04	-	-	-
	GO:0019838 growth factor binding	-	3.70E-03	-	-	-	-	-	7.70E-03	4.74E-02	-
	GO:0008092 cytoskeletal protein binding	5.70E-04	-	1.39E-02	-	-	-	-	-	-	-
hsa-mir-30a	GO:0003676 nucleic acid binding	-	-	-	9.80E-03	-	-	-	6.20E-04	-	-
	GO:0003723 RNA binding	-	-	-	1.21E-02	-	-	-	9.09E-07	-	-
	GO:0019899 enzyme binding	-	-	-	1.94E-02	-	-	-	-	4.60E-03	-
	GO:1901363 heterocyclic compound binding	-	-	-	5.90E-03	-	-	-	-	-	-
	GO:0097159 organic cyclic compound binding	-	-	-	5.90E-03	-	-	-	-	-	-
hsa-let-7i	GO:0051575 5'-deoxyribose-5-phosphate lyase activity	-	-	-	-	2.80E-03	-	-	-	-	-
	GO:0008134 transcription factor binding	-	-	-	-	-	-	-	2.84E-02	-	-
	GO:0140297 DNA-binding transcription factor binding	-	-	-	-	-	-	-	4.46E-02	-	-
	GO:0140110 transcription regulator activity	-	-	-	-	-	-	-	4.46E-02	-	-
	GO:0042826 histone deacetylase binding	-	-	-	-	-	-	-	4.46E-02	-	-
hsa-mir-24	GO:0035173 histone kinase activity	2.30E-05	2.34E-05	-	-	2.36E-05	-	2.17E-05	4.17E-07	-	2.70E-05
	GO:0140097 catalytic activity, acting on DNA	5.20E-04	7.50E-03	-	-	-	-	-	5.05E-05	-	-
	GO:1901363 heterocyclic compound binding	1.40E-03	7.50E-03	-	-	-	-	-	2.80E-04	-	-
	GO:0005515 protein binding	-	-	2.10E-02	-	5.30E-03	6.80E-04	-	-	-	-
	GO:0003684 damaged DNA binding	2.90E-04	-	-	-	-	-	-	1.46E-05	-	-
hsa-mir-95	GO:1901363 heterocyclic compound binding	-	-	3.00E-03	-	-	-	4.04E-02	1.03E-02	-	-
	GO:0140110 transcription regulator activity	-	-	3.00E-03	-	-	-	4.04E-02	7.70E-03	-	-
	GO:0097159 organic cyclic compound binding	-	-	3.00E-03	-	-	-	4.04E-02	1.03E-02	-	-
	GO:0003676 nucleic acid binding	-	-	3.00E-03	-	-	-	3.42E-02	5.20E-03	-	-
	GO:0043565 sequence-specific DNA binding	-	-	3.00E-03	-	-	-	-	1.03E-02	-	-

REFERENCES

Altman, N. S. (1992). An introduction to kernel and nearest-neighbor nonparametric regression. *The American Statistician* 46, 175–185

Bennasar, M., Hicks, Y., and Setchi, R. (2015). Feature selection using joint mutual information maximisation. *Expert Systems with Applications* 42, 8520–8532

Breiman, L. (2005). Random Forests. *Machine Learning* 45, 5–32

Brown, G., Pocock, A., Zhao, M., and Lujan, M. (2012a). Conditional likelihood maximisation: a unifying framework for information theoretic feature selection. *The Journal of Machine Learning Research* 13, 27–66

Brown, G., Pocock, A., Zhao, M. J., and Lujan, M. (2012b). Conditional Likelihood Maximisation: A Unifying Framework for Information Theoretic Feature Selection. *Bioinformatics* 13, 27–66

Cortes, C. and Vapnik, V. (1995). Support-vector networks. *Machine Learning* 20, 273–297. doi:10.1007/BF00994018

Fleuret, F. (2004). Fast binary feature selection with conditional mutual information. *Journal of Machine Learning Research* 5, 1531–1555. 00621

George, H. and Langley, J. P. (1995). Estimating Continuous Distributions in Bayesian Classifiers. *Proceedings of the Eleventh Conference on Uncertainty in Artificial Intelligence* 69, 338–345

Hsu, C. W. and Lin, C. J. (2002). A comparison of methods for multiclass support vector machines. *IEEE Transactions on Neural Networks* 13, 415–425

Jakulin, A. (2005). Machine learning based on attribute interactions. *Univerza v Ljubljani*

Peng, H., Long, F., and Ding, C. (2005). Feature selection based on mutual information: criteria of max-dependency, max-relevance, and min-redundancy. *IEEE Transactions on Pattern Analysis and Machine Intelligence* 27, 1226–1238

Quinlan, J. R. (1986). Induction of decision trees. *Machine Learning* 1, 81–106

Quinlan, J. R. (1993). *C4.5: Programs for Machine Learning* (San Francisco, CA, USA: Morgan Kaufmann Publishers Inc.)

Troyanskaya, O. G., Garber, M. E., Brown, P. O., Botstein, D., and Altman, R. B. (2002). Nonparametric methods for identifying differentially expressed genes in microarray data. *Bioinformatics* 18, 1454–1461

Table S5. *Five* significant GO Cellular Component for each selected 17 miRNAs in ten diverse cancer types

miRNA	GO-Cellular Component	FDR corrected *p-value*									
		BLCA	BRCA	COAD	GBM	HNSC	KIRC	LUAD	LUSC	OV	UCEC
hsa-mir-205	GO:0043227 membrane-bounded organelle	-	3.22E-07	-	3.30E-07	-	6.10E-03	2.70E-03	1.36E-02	9.28E-06	1.10E-05
	GO:0043231 intracellular membrane-bounded organelle	-	1.31E-05	-	3.30E-07	-	6.10E-03	3.30E-03	1.52E-02	5.34E-06	-
	GO:0044424 intracellular part	-	1.50E-04	3.76E-02	3.30E-07	-	1.81E-02	2.60E-03	-	2.20E-04	-
	GO:0043229 intracellular organelle	-	2.17E-05	-	3.30E-07	-	1.81E-02	-	-	-	1.80E-04
	GO:0044446 intracellular organelle part	-	6.60E-04	-	-	-	-	-	-	-	1.80E-04
hsa-mir-10a	GO:0044446 intracellular organelle part	5.70E-03	-	2.70E-02	-	1.61E-05	2.58E-02	-	3.57E-06	-	-
	GO:0044428 nuclear part	5.70E-03	1.78E-08	-	-	-	-	1.50E-04	2.43E-08	2.56E-05	-
	GO:0044424 intracellular part	5.70E-03	-	-	1.90E-03	-	-	2.00E-03	-	4.79E-05	1.09E-02
	GO:0070013 intracellular organelle lumen	5.70E-03	6.00E-07	-	-	-	3.00E-03	-	3.57E-06	-	-
	GO:0031981 nuclear lumen	-	6.85E-09	-	-	-	-	5.20E-04	2.43E-08	7.62E-05	-
hsa-mir-196b	GO:0005654 nucleoplasm	3.50E-03	4.20E-03	4.23E-05	2.00E-03	4.80E-03	1.20E-03	2.86E-02	1.22E-02	6.50E-04	1.80E-03
	GO:0032991 protein-containing complex	7.40E-03	4.41E-06	4.23E-05	1.30E-04	3.40E-04	1.20E-03	3.40E-04	9.50E-04	1.29E-06	4.03E-05
	GO:0070013 intracellular organelle lumen	7.40E-03	4.00E-03	1.00E-03	1.60E-03	3.80E-03	1.20E-03	-	-	1.00E-03	5.90E-03
	GO:0044428 nuclear part	7.40E-03	-	1.00E-03	7.10E-03	-	-	3.44E-02	-	1.30E-03	5.90E-03
	GO:0031981 nuclear lumen	7.40E-03	6.80E-03	-	-	6.50E-03	1.20E-03	-	1.22E-02	1.30E-03	-
hsa-mir-10b	GO:0044428 nuclear part	4.20E-03	5.19E-06	-	-	-	8.00E-03	7.50E-04	-	3.20E-02	-
	GO:0044424 intracellular part	2.70E-03	-	1.42E-02	1.81E-07	-	-	-	-	3.20E-02	-
	GO:0044464 cell part	2.70E-03	-	4.90E-03	-	1.80E-04	-	-	-	-	-
	GO:0044446 intracellular organelle part	-	2.17E-05	-	5.81E-08	-	-	-	-	3.20E-02	-
	GO:0044427 chromosomal part	-	2.17E-05	-	-	1.20E-04	3.56E-02	-	-	-	-
hsa-mir-375	GO:0044464 cell part	-	2.96E-02	-	1.80E-04	-	-	2.30E-04	-	3.68E-02	-
	GO:0044424 intracellular part	-	-	-	1.80E-04	3.50E-04	-	4.80E-04	-	8.60E-03	-
	GO:0043226 organelle	-	-	-	3.39E-06	3.50E-04	2.10E-03	-	-	-	-
	GO:0043229 intracellular organelle	-	-	-	5.48E-06	3.50E-04	2.10E-03	-	-	-	-
	GO:0012505 endomembrane system	-	-	1.20E-03	-	5.54E-05	-	-	-	-	-
hsa-mir-143	GO:0043231 intracellular membrane-bounded organelle	2.50E-04	-	1.11E-02	4.79E-02	-	-	-	-	5.92E-05	-
	GO:0044446 intracellular organelle part	-	6.70E-04	1.11E-02	-	-	-	3.30E-04	-	-	-
	GO:0044428 nuclear part	2.90E-04	-	1.04E-02	-	-	-	-	-	-	-
	GO:0044444 cytoplasmic part	-	6.57E-05	-	-	-	-	1.20E-04	-	-	-
	GO:0005737 cytoplasm	-	3.20E-04	-	-	-	-	1.60E-04	-	-	-
hsa-let-7c	GO:0070013 intracellular organelle lumen	1.50E-04	5.57E-08	3.10E-04	1.60E-04	6.55E-06	1.60E-02	-	3.18E-07	-	-
	GO:0043231 intracellular membrane-bounded organelle	1.50E-04	6.53E-08	1.50E-03	-	-	-	3.67E-05	-	1.40E-04	4.70E-04
	GO:0032991 protein-containing complex	1.45E-05	5.70E-08	-	9.40E-04	6.55E-06	-	-	3.28E-08	-	-
	GO:0005654 nucleoplasm	-	-	2.70E-04	-	8.49E-06	2.39E-02	2.73E-05	-	1.40E-04	-
	GO:0044446 intracellular organelle part	-	6.11E-10	8.00E-04	-	-	-	6.40E-06	4.69E-07	-	-
hsa-mir-107	GO:0044464 cell part	-	1.50E-04	-	6.80E-04	2.50E-03	1.40E-04	7.10E-04	5.00E-03	6.36E-05	-
	GO:0044424 intracellular part	-	8.00E-04	1.35E-07	6.80E-04	2.50E-03	1.01E-06	1.69E-02	-	4.90E-03	-
	GO:0044446 intracellular organelle part	8.50E-03	-	1.42E-05	6.80E-04	-	5.94E-05	1.69E-02	-	5.40E-03	-
	GO:0043226 organelle	-	-	5.05E-06	6.80E-04	3.40E-03	1.40E-04	-	-	-	-
	GO:0005634 nucleus	2.70E-03	1.50E-04	-	-	-	-	1.17E-02	-	-	-
hsa-mir-378	GO:0043231 intracellular membrane-bounded organelle	3.45E-07	4.71E-07	-	-	-	-	1.86E-06	4.27E-06	-	-
	GO:0043227 membrane-bounded organelle	-	2.92E-07	-	-	2.60E-04	-	2.49E-06	1.01E-05	-	-
	GO:0070013 intracellular organelle lumen	3.45E-07	-	-	-	-	-	1.36E-05	1.97E-06	-	-
	GO:0044424 intracellular part	-	-	-	-	-	2.20E-03	1.12E-05	1.97E-06	-	-
	GO:0043229 intracellular organelle	-	3.30E-07	-	-	2.60E-04	-	-	-	-	-
hsa-mir-133a	GO:0044424 intracellular part	3.40E-03	3.50E-03	2.90E-03	5.20E-05	4.40E-04	1.30E-04	2.50E-03	2.60E-03	2.90E-04	2.10E-04
	GO:0043229 intracellular organelle	1.50E-03	2.80E-04	2.90E-03	7.36E-06	2.10E-04	3.49E-06	8.80E-04	-	8.22E-05	2.10E-04
	GO:0005622 intracellular	1.90E-03	3.50E-03	2.90E-03	5.20E-05	-	5.05E-05	1.30E-03	2.60E-03	-	-
	GO:0044446 intracellular organelle part	3.40E-03	-	-	2.16E-05	4.40E-04	-	-	-	2.90E-04	2.10E-04
	GO:0044433 cytoplasmic vesicle part	-	8.10E-03	-	-	4.40E-04	-	2.38E-02	-	5.80E-04	1.12E-06
hsa-mir-1	GO:0044428 nuclear part	1.35E-12	-	-	-	-	5.50E-03	2.41E-09	1.47E-07	-	9.09E-06
	GO:0031981 nuclear lumen	7.11E-12	-	-	-	-	5.50E-03	1.53E-08	-	-	1.59E-05
	GO:0043226 organelle	8.06E-12	-	-	3.48E-02	-	5.50E-03	-	-	2.40E-03	-
	GO:0044446 intracellular organelle part	2.17E-12	3.93E-06	1.10E-03	-	-	-	-	-	-	-
	GO:0070013 intracellular organelle lumen	8.06E-12	-	-	-	-	5.50E-03	1.69E-10	-	-	-
hsa-mir-30c	GO:0044424 intracellular part	4.47E-06	-	5.48E-06	3.10E-06	3.50E-04	-	2.89E-07	2.30E-04	7.00E-04	-
	GO:0005829 cytosol	4.47E-06	1.20E-03	-	-	3.50E-04	1.34E-02	7.13E-08	4.73E-05	3.40E-04	-
	GO:0044444 cytoplasmic part	1.06E-05	1.20E-03	1.05E-05	-	3.50E-04	5.60E-03	-	-	6.83E-06	-
	GO:0005737 cytoplasm	-	-	6.43E-06	-	3.50E-04	5.60E-03	2.89E-07	-	1.33E-06	-
	GO:0044446 intracellular organelle part	1.12E-05	-	-	-	-	-	1.67E-07	2.30E-04	-	3.32E-07
hsa-mir-16	GO:0071944 cell periphery	4.60E-03	-	-	1.30E-03	-	-	9.04E-05	2.23E-08	-	-
	GO:0044444 cytoplasmic part	-	-	3.35E-02	-	4.80E-03	1.83E-07	-	-	-	-
	GO:0005737 cytoplasm	-	-	-	-	2.00E-04	-	-	-	3.18E-02	-
	GO:0044424 intracellular part	-	-	-	-	1.41E-02	-	-	-	3.18E-02	-
	GO:0005886 plasma membrane	-	-	-	-	-	-	5.20E-04	7.03E-08	-	-
hsa-mir-30a	GO:0044446 intracellular organelle part	-	2.70E-05	1.97E-07	3.18E-05	-	9.00E-03	2.23E-06	-	1.59E-05	-
	GO:0005622 intracellular	-	5.41E-06	-	-	9.70E-04	9.00E-03	-	-	4.29E-06	-
	GO:0070013 intracellular organelle lumen	-	-	1.35E-06	5.10E-05	-	-	9.07E-06	3.23E-13	-	-
	GO:0031981 nuclear lumen	-	-	1.35E-06	1.59E-06	-	-	4.55E-06	3.23E-13	-	-
	GO:0044424 intracellular part	-	5.41E-06	-	-	-	-	-	-	4.29E-06	3.16E-06
hsa-let-7i	GO:0044444 cytoplasmic part	-	-	3.67E-02	-	-	4.70E-03	-	-	-	3.20E-04
	GO:0005737 cytoplasm	-	-	3.67E-02	-	-	9.10E-04	-	-	-	8.60E-04
	GO:0044424 intracellular part	-	-	-	-	-	7.40E-03	1.15E-02	-	2.26E-02	-
	GO:0098805 whole membrane	-	-	3.67E-02	-	-	-	-	-	-	-
	GO:0044429 mitochondrial part	-	-	-	-	-	6.80E-03	-	-	-	-
hsa-mir-24	GO:0005654 nucleoplasm	3.33E-10	4.80E-08	-	-	7.46E-06	-	2.21E-05	1.07E-09	-	5.23E-05
	GO:0070013 intracellular organelle lumen	2.97E-09	4.80E-08	-	-	1.95E-06	-	5.55E-07	4.34E-10	1.35E-02	-
	GO:0044428 nuclear part	8.95E-10	-	-	-	7.46E-06	-	-	4.34E-10	1.35E-02	5.23E-05
	GO:0031981 nuclear lumen	8.95E-10	8.57E-08	-	-	-	-	-	4.34E-10	-	7.22E-05
	GO:0044446 intracellular organelle part	-	-	-	-	1.95E-06	-	1.64E-06	-	1.35E-02	3.88E-05
hsa-mir-95	GO:0044424 intracellular part	1.48E-02	-	5.40E-03	8.50E-03	8.10E-03	2.20E-04	2.16E-02	1.62E-02	7.70E-04	7.80E-03
	GO:0043229 intracellular organelle	1.48E-02	-	5.40E-03	1.90E-04	8.10E-03	1.90E-04	1.44E-02	1.90E-03	1.80E-04	1.09E-02
	GO:0043226 organelle	1.48E-02	-	5.40E-03	1.90E-04	8.10E-03	-	1.44E-02	1.90E-03	1.80E-04	-
	GO:0005634 nucleus	1.48E-02	-	5.40E-03	-	-	-	2.16E-02	2.00E-03	5.50E-03	1.09E-02
	GO:0005622 intracellular	-	-	-	-	-	2.20E-04	2.16E-02	1.62E-02	-	7.80E-03

Table S6. Additional experiment results

**Sr. No.**	**URL for additional files**
1.	Association of selected 17 miRNAs and their targets in ten diverse cancer types
2.	Summary of selected miRNAs and their targets in ten diverse cancer types
3.	KEGG Pathway Analysis
4.	GO Biological Process Analysis
5.	GO Molecular Function Analysis
6.	GO Cellular Component Analysis
7.	Protein Protein Interaction Analysis
